# Circular Polydiketoenamine Elastomers with Exceptional Creep Resistance via Multivalent Cross-Linker Design

**DOI:** 10.1021/acscentsci.3c01096

**Published:** 2023-11-17

**Authors:** Eric A. Dailing, Pawan Khanal, Alexander R. Epstein, Jeremy Demarteau, Kristin A. Persson, Brett A. Helms

**Affiliations:** † Molecular Foundry Lawrence Berkeley National Laboratory 1 Cyclotron Road, Berkeley, California 94270, United States; ‡ Materials Sciences and Engineering University of California, Berkeley Berkeley, California 94720, United States; § Materials Sciences Division Lawrence Berkeley National Laboratory 1 Cyclotron Road, Berkeley, California 94270, United States

## Abstract

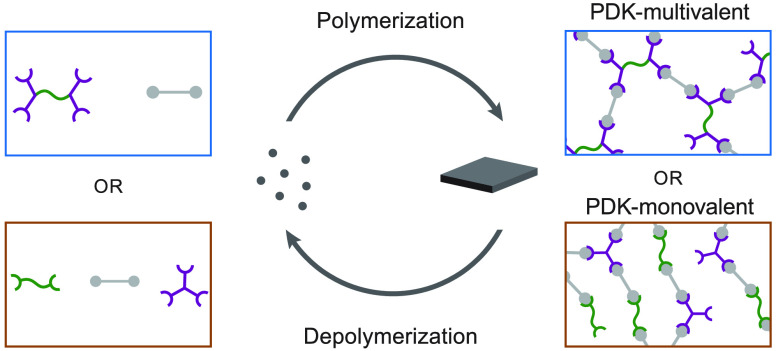

Elastomers are widely used in textiles, foam, and rubber, yet they are rarely recycled due to the difficulty in deconstructing polymer chains to reusable monomers. Introducing reversible bonds in these materials offers prospects for improving their circularity; however, concomitant bond exchange permits creep, which is undesirable. Here, we show how to architect dynamic covalent polydiketoenamine (PDK) elastomers prepared from polyetheramine and triketone monomers, not only for energy-efficient circularity, but also for outstanding creep resistance at high temperature. By appending polytopic cross-linking functionality at the chain ends of flexible polyetheramines, we reduced creep from >200% to less than 1%, relative to monotopic controls, producing mechanically robust and stable elastomers and carbon-reinforced rubbers that are readily depolymerized to pure monomer in high yield. We also found that the multivalent chain end was essential for ensuring complete PDK deconstruction. Mapping reaction coordinates in energy and space across a range of potential conformations reveals the underpinnings of this behavior, which involves preorganization of the transition state for diketoenamine bond acidolysis when a tertiary amine is also nearby.

## Abstract 2

Valency of soft segments of polydiketoenamine elastomer networks dictates chemical recycling and creep resistance. Multivalent chain ends enable circularity for high-value polymers that are conventionally difficult to recycle.

## Introduction

Cross-linked elastomers and rubbers have broad commercial and industrial uses due to their thermal, chemical, and mechanical stabilities. These stabilities make deconstructing them to their original monomers a persistent challenge, yet important for a circular plastics economy.^1,2^ Elastomer networks featuring reversible bonds open the door to chemical recycling.^3−9^ However, most fail to close the loop upon deconstruction: more often, chemical recycling returns fragments of the network rather than monomers.^10−14^ Chemical recycling to monomer has been most successful when networks are cross-linked using dynamic covalent bonds that can be cleaved solvolytically.^15−21^ However, bond exchange reactions inherent to dynamic covalent elastomers also promote creep and the materials exhibit poor mechanical stability in load-bearing environments, particularly at elevated temperature.^22^ Controlling viscous flow to minimize or eliminate creep in future generations of circular elastomers therefore requires more careful consideration, not only of the reversible bond but also of the network architecture.^23−26^ Ideally, short-range bond exchange kinetics and long-range viscoelastic flow can be uncoupled.

**Figure 1:**
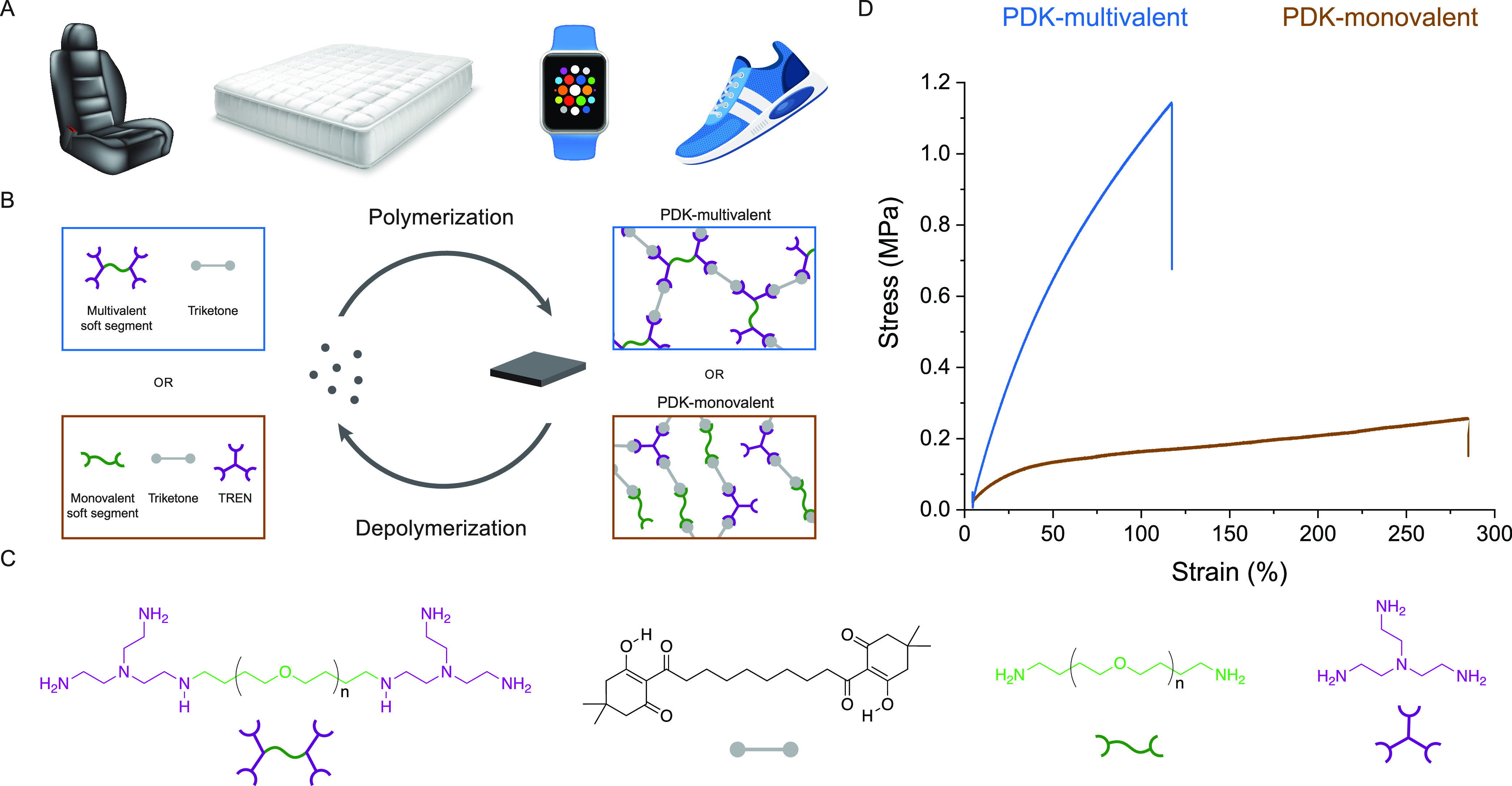
PDK elastomer formulation and stress–strain behavior. (A) Examples of commercial products that incorporate cross-linked elastomeric components that are challenging to recover and recycle. (B) Schematics of macromer, monomer, and corresponding polymer network structure for PDK-multivalent and PDK-monovalent. (C) Macromer and monomer structures for multivalent soft segment, poly(tetrahydrofuran)-bis-tris-2(aminoethyl)amine (pTHF-bis-TREN); triketone, 2,2′-decanedioyl-bis(5,5-dimethylcyclohexane-1,3-dione) (TK-10); monovalent soft segment, poly(tetrahydrofuran)-diamine (pTHF-diamine); TREN, tris-2(aminoethyl)amine. (D) Stress–strain plots for PDK-multivalent (strain at break = 104%, tensile strength at break = 1.14 MPa, toughness = 0.785 MJ m^–3^) and PDK-monovalent (strain at break = 268%, tensile strength at break = 0.257 MPa, toughness = 0.499 MJ m^–3^).

Here, we show how to architect circular polydiketoenamine (PDK) elastomers and carbon black reinforced PDK rubbers to resist creep by tailoring the valency at cross-linking sites associated with flexible polyetheramine segments within the network (Figure 1). Notable in our designs, diketoenamine bonds retain their ability to participate in short-range bond exchange, enabling thermoforming during manufacturing and stress relaxation upon strain; however, viscoelastic flow over longer length scales is disfavored due to the multiplicity of anchor points to the network, which render the elastomers exceptionally resistant to creep, even at high temperature. Interestingly, we found that the end-group structure of the polyetheramine soft segments, herein referred to as macromers, also dictated the rate of PDK depolymerization during chemical recycling: whereas monovalent polyetheramine macromers producing creep-susceptible PDK elastomers were slow to depolymerize, multivalent polyetheramine macromers producing creep-resistant PDK elastomers were completely depolymerized within 24 h. This enabled facile recovery of the multivalent polyetheramine macromer and triketone monomer in high yields with high purity, permitting their reuse in subsequent manufacturing cycles. To understand this behavior, we developed a theoretical framework to explore reactive conformations along the reaction coordinate in PDK hydrolysis. In doing so, we identified the key role played by a proximal ionized amine, exclusive to the multivalent polyetheramine end group, that preorganizes the transition state associated with the rate-limiting step, lowering the standard free energy of activation (Δ*G*^‡^) by 13 kJ mol^–1^ for diketoenamine hydrolysis in strong acid. Transition-state preorganization emerges with due importance in achieving high efficiency and low-carbon intensity in PDK chemical recycling. This work advances macromolecular design of elastomeric soft segments in chemically recyclable dynamic covalent thermosets that simultaneously enable mechanical stability and depolymerization to monomer. Given the vast chemical space available to both polyetheramine and triketone monomers, we envision the knowledge gleaned in this work can be exploited for codesigning future elastomers and rubbers on the basis of both performance and circularity in chemical recycling.

## Results and Discussion

### PDK Elastomer Synthesis and Formulation

PDK resins are created from a wide variety of polytopic triketone and amine macromers and/or monomers via spontaneous polycondensation (i.e., click) reactions.^27^ Until now, our navigation of monomer chemical space has produced exclusively rigid vitrimers, whose glass transition temperatures (*T*_g_) of 70–150 °C prioritized their use to applications requiring strength and structural integrity in that working temperature range.^27−29^ We hypothesized that, by incorporating flexible amine macromers into the network, it would be possible to explore new regions of chemical space to seek the subambient temperature *T*_g_ and impart elasticity to the network architecture. If successful, the circularity afforded by PDK materials would extend to a broader range of useful polymeric materials, particularly elastomers and reinforced rubbers (Figure 1A).

The manner in which flexible amine macromers should be integrated into PDK networks is nontrivial, given the tendency of elastomers to creep in polymer networks cross-linked with dynamic covalent bonds.^22,30,31^ We hypothesized that, by increasing the valency of amine end groups in the flexible amine macromer, it would be possible for PDK networks to retain their ability to engage in short-range bond exchange reactions; however, long-range viscoelastic flow responsible for creep would be disfavored due to the large number of anchor points of the amine macromer to the larger network architecture. To test this hypothesis, we designed and synthesized flexible polyetheramine macromers with multivalent amine end groups. Specifically, we transformed the chain ends of polytetrahydrofuran diol (pTHF-diol) to the corresponding mesylates prior to reaction with tris(2-aminoethylamine) (TREN) to obtain the target macromer, pTHF-bis-TREN. We then prepared cross-linked PDK elastomers from pTHF-bis-TREN and a triketone monomer (TK-10) separately synthesized from dimedone and sebacic acid (Figure 1B,C). The amine-to-triketone molar ratio was 1.3:1, and solid samples of these multivalent PDK elastomers (PDK-multivalent) were obtained within 30 s of polymerization at 60 °C in THF. As a control, to understand the impact of PDK network architecture and macromer end-group structure on thermomechanical properties and efficiency of recycling circularity, we also prepared cross-linked PDK elastomers (PDK-monovalent) from pTHF-diamine (i.e., a flexible polyether amine macromer with monovalent amine end groups), TREN as the cross-linker, and TK-10. We matched the pTHF weight fraction between the two formulations as closely as possible (75% w/w for PDK-monovalent vs 76% w/w for PDK-multivalent) and again set the total amine-to-triketone molar ratio to 1.3:1 for PDK-monovalent. Polymerized samples were easily remolded into defined shapes by pressing in Teflon molds at 150 °C and 60 psi for 5 min (Figure S1). This method produced a high gel fraction for the monovalent and multivalent formulations (94.1 and 96.3%, respectively). We then performed tensile stress–strain measurements as an initial investigation of mechanical properties (Figure 1D). PDK-multivalent had a lower elongation at break relative to PDK-monovalent (104% vs 268%) yet maintained significantly higher tensile strength (1.14 MPa vs 0.257 MPa) and toughness (0.785 MJ m^–3^ vs 0.499 MJ m^–3^). These results suggested a strong dependence between network architecture and properties in PDK elastomers, motivating a deeper investigation into their rheological behavior.

### Soft Segment Chain-End Structure Determines Cross-Linking and Stress Relaxation in PDK Elastomers

**Figure 2:**
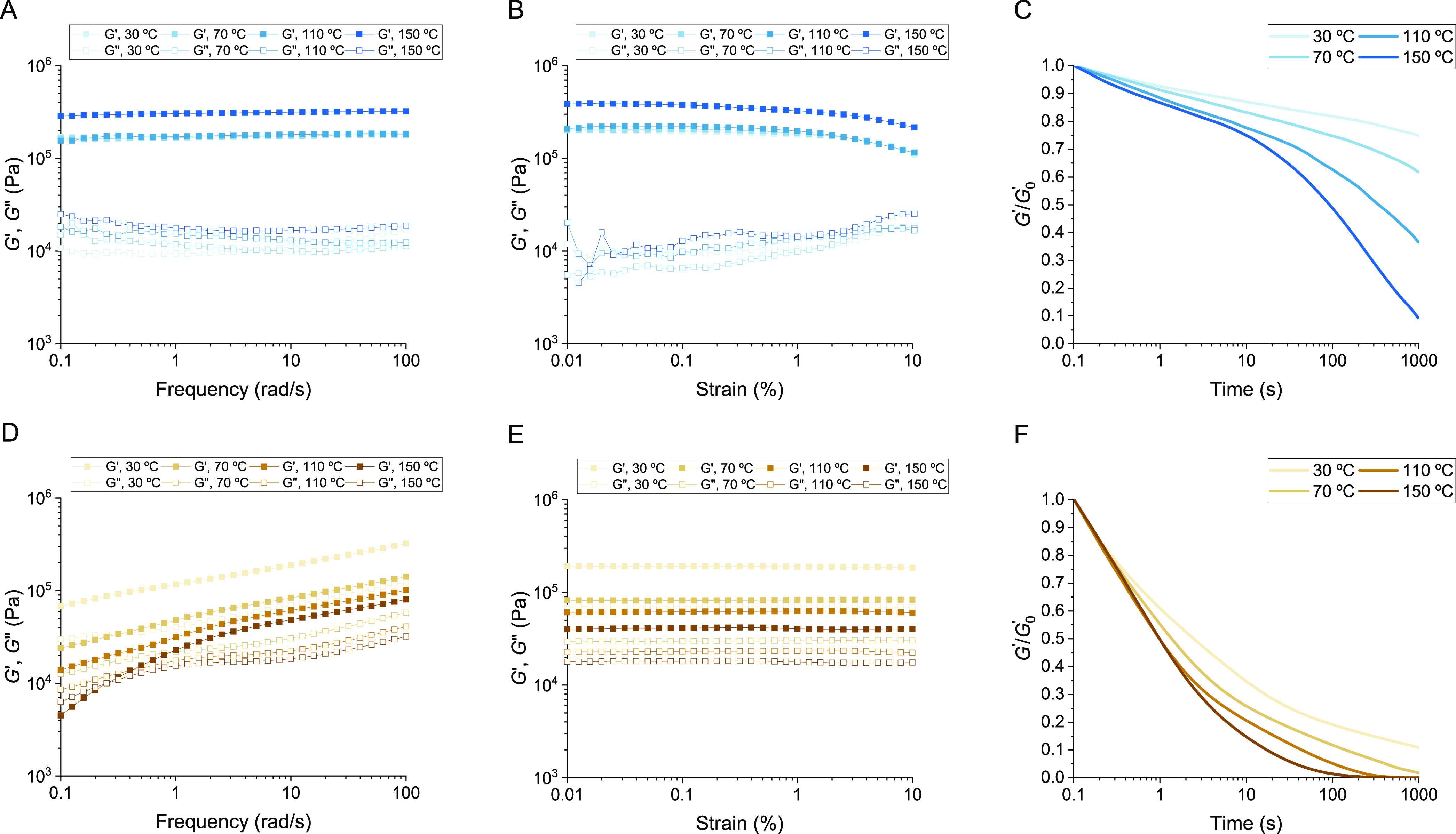
Rheological characterization of PDK elastomers. (A) Frequency sweep, (B) amplitude sweep, and (C) stress relaxation measurements for PDK-multivalent elastomers. (D) Frequency sweep, (E) amplitude sweep, and (F) stress relaxation measurements for PDK-monovalent elastomers.

To test the second part of our hypothesis, pertaining to long-range viscoelastic flow, we performed a series of rheology experiments to reveal how amine macromer valency within the PDK network architecture dictates the modulus and transient flow behavior of the materials (Figure 2). To study the interactions between the network and reinforcing fillers, we further carried out each PDK polycondensation in the presence of 0.5 wt % carbon black, which produced black-pigmented carbon-reinforced PDK rubbers with essentially quantitative incorporation of the filler. Similar to the unfilled PDK formulations, the carbon-reinforced rubbers were amenable to thermoforming at 150 °C and 60 psi, producing samples that conformed to Teflon molds after 5 min of processing (Figure S1). This confirmed part of our initial hypothesis in that short-range diketoenamine bond exchange remained feasible for all materials in this study. We carried out these experiments between 30 and 150 °C, which is above the temperature of all thermal transitions in both PDK elastomers and rubbers, as observed by differential scanning calorimetry (DSC; Figures S2 and S3).

Frequency sweep measurements quantifying the storage modulus (*G*′) and loss modulus (*G*″) for PDK-multivalent elastomers showed no crossover point in *G*′/*G*″ over the measured frequency range and minimal frequency dependence on the modulus (Figure 2A). The modulus of PDK-multivalent elastomers was 200 kPa at 30 °C and increased slightly to 210 kPa at 110 °C. Notably, at 150 °C, the modulus nearly doubled to 390 kPa (Figure 2B). Our observation of *G*′ increasing with temperature is consistent with previous reports for polymer networks that engage in associative bond exchange.^32,33^ Moreover, we note that this phenomenon in well-described by rubbery elasticity theory,^34^ which states that the force (*f*) required to deform a network is related to temperature and the change in entropy with sample length (*L*) through the equation
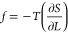
1

Since conformational entropy decreases when a polymer network is deformed, *f* becomes a positive quantity. We further relate temperature (*T*) to the storage modulus (*G*) as
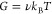
2where ν is network strands per unit volume and *k*_B_ is Boltzmann’s constant, demonstrating the direct proportionality between *G* and *T*.^34^ Surprisingly, however, we observed the opposite trend with PDK-monovalent elastomers, for which *G*′ decreased monotonically with temperature. Compared to the modulus for PDK-multivalent elastomers, the modulus for PDK-monovalent elastomers showed a strong frequency dependence, which indicates relatively shorter network relaxation times due to higher chain mobility (Figure 2D).^35,36^ We also noted a *G*′/*G*″ crossover at 150 °C, further confirming that, at elevated temperature, chain mobility is high enough to permit long-range viscous flow.

Critical to understanding this trend is the observation that the modulus for PDK-monovalent elastomers at 30 °C (190 kPa) (Figure 2E) is comparable to that for PDK-multivalent elastomers (200 kPa) (Figure 2B) at the same temperature, despite PDK-monovalent elastomers containing less TREN as a cross-linker. This suggests that, at relatively low temperature, noncovalent entanglements manifest in PDK-monovalent networks that increase the apparent cross-link density (ν). We calculated the cross-linking density for both formulations from the equation
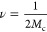
3where
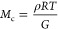
4Here, ρ is bulk density, *R* is the gas constant, *T* is absolute temperature, and *G* is the shear storage modulus at 30 °C. We obtain ν = 3.32 × 10^–5^ mol g^–1^ for PDK-monovalent and ν = 3.59 × 10^–5^ mol g^–1^ for PDK-multivalent, confirming comparable cross-linking densities for both formulations.

As the temperature increases, excess amines can participate in diketoenamine bond exchange reactions to disentangle the linear segments within the network, allowing it to reach an equilibrium state with comparatively lower ν and thus a lower observed modulus. By contrast, PDK-multivalent elastomers have a higher density of covalent cross-links by virtue of the pTHF-bis-TREN multivalent chain-end structure, which appears to dominate over any loss of noncovalent network entanglements associated with diketoenamine bond exchange.

We observed further evidence that network reorganization is facile for PDK-monovalent elastomers by comparison with PDK-multivalent elastomers in the stress relaxation data for both (Figure 2C,F). Stress relaxation in PDK-multivalent elastomers did not follow a simple exponential decay, suggesting far more complex behavior associated with its relaxation than conventional models account for, e.g., extracting the activation energy (Arrhenius) or standard free energy of activation (Eyring) for bond exchange.^34,37^ Instead, we compared the relative rates of relaxation between PDK-multivalent and PDK-monovalent elastomers, noting that the characteristic time for PDK-monovalent to relax to a reduced modulus of e^–1^ is greater than 2 orders of magnitude faster than that for PDK-multivalent. We observe a two-step relaxation process in PDK-multivalent networks representing the presence of relatively fast and slow exchanging network segments,^38^ compared to a single-step relaxation for PDK-monovalent. This is likely due to the pTHF-bis-TREN end group structure, wherein stress relaxation in PDK-multivalent networks requires rearrangement of at least three bonds while PDK-monovalent requires only one bond to rearrange. PDK-monovalent networks feature relatively long linear chains with exchangeable diketoenamine bonds along the backbone which further enables more facile associative bond exchange, compared to PDK-multivalent networks in which all PDK bonds and excess amines are confined to the cross-linking points. The temperature-dependent data for *G*′ and *G*″ indicate a preservation of noncovalent and covalent cross-linking densities in PDK-multivalent elastomers and an apparent lowering of the cross-linking density in PDK-monovalent elastomers. It follows that stress relaxation in PDK-monovalent elastomers is concomitant with a decrease in the noncovalent contribution to network cross-linking density, since covalent cross-linking density is constant. Moreover, this occurs only in PDK-monovalent elastomers because bond exchange irreversibly reduces linear chain entanglement under the applied strain. Since the presence of physical entanglements in covalently cross-linked rubbery polymers is known to increase fracture toughness,^39,40^ we can couple the stress relaxation observations with the results in Figure 1D and conclude that permanent entanglements in PDK-monovalent are unlikely to persist on the time scale of these experiments. Taken together, these observations demonstrate that covalently confining the cross-linking points to the soft segment chain ends produces slower terminal relaxation in PDK-multivalent elastomers, while physical entanglements in PDK-monovalent elastomers are rapidly lost during bond exchange.

### Carbon Black Produces a Reinforcing Effect in PDK Elastomers

**Figure 3:**
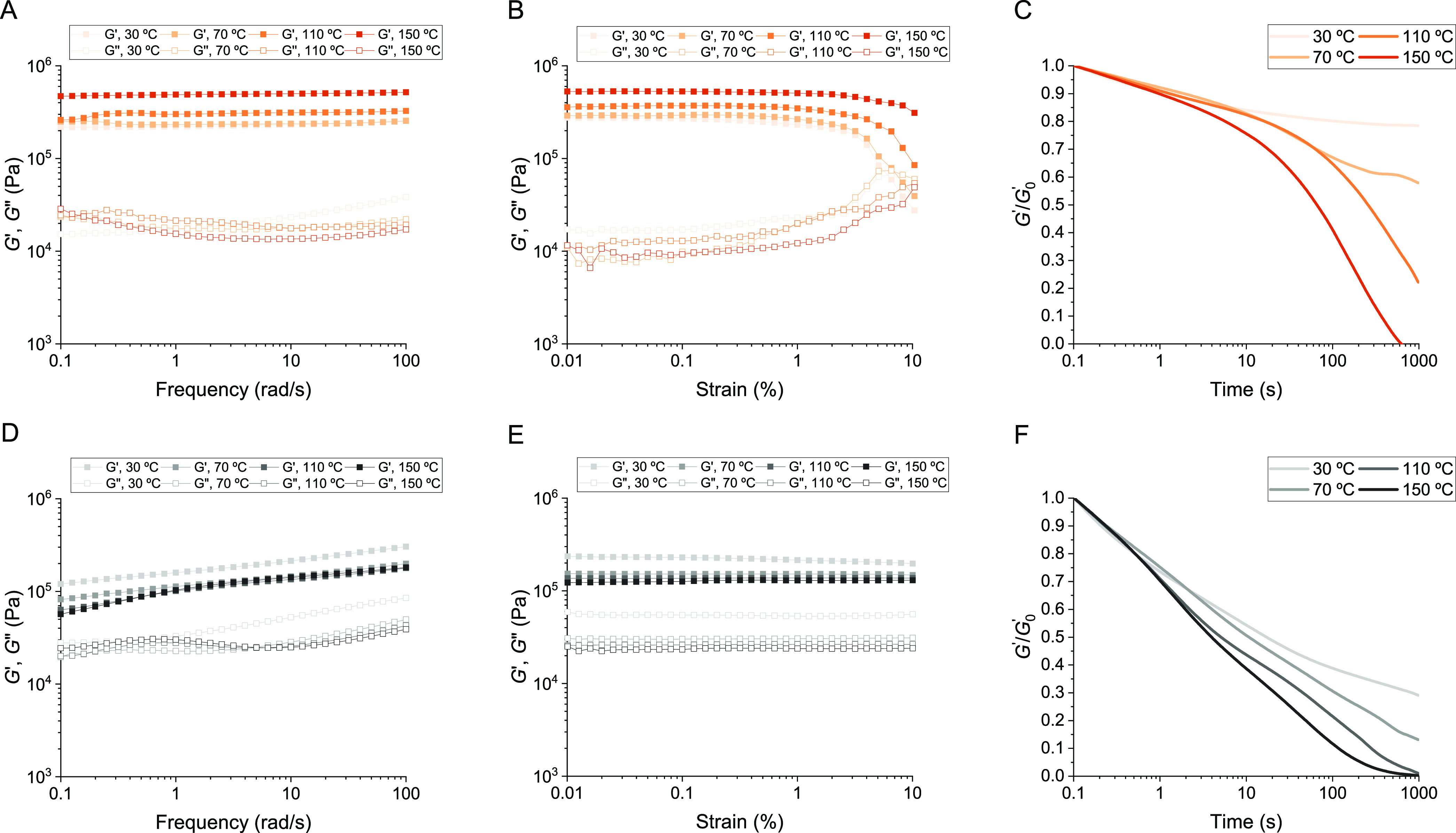
Rheological characterization of carbon black reinforced PDK elastomers. (A) Frequency sweep, (B) amplitude sweep, and (C) stress relaxation measurements for PDK-multivalent containing 0.5 wt % carbon black. (D) Frequency sweep, (E) amplitude sweep, and (F) stress relaxation measurements for PDK-monovalent containing 0.5 wt % carbon black.

We then examined how carbon black as a filler affects PDK elastomer rheology. Carbon black is widely used to reinforce commercial rubbers and was easily dispersed into the PDK materials with no observable effect on polymerization. FTIR spectra for both PDK-multivalent and PDK-monovalent showed no changes in vibrational modes when carbon black was added (Figures S4 and S5). Yet, we observed a 40% increase in storage modulus when incorporating only 0.5 wt % carbon black into PDK-multivalent elastomers (Figure 3A,B), possibly due to the formation of a bound rubber layer at the elastomer–carbon black interface.^41,42^ Because of the unique architecture of PDK-multivalent elastomers, interactions with carbon black are more likely to involve the pTHF segments, since potentially coordinating amine functionalities are most often found at sterically encumbered sites within the network. In stark contrast, coordinating amine functionality in PDK-monovalent elastomers may be found throughout the network, thus promoting adsorption on that basis to a greater degree. Consequently, the reduction in chain mobility produced a larger reinforcing effect for PDK-monovalent elastomers, resulting in a reduced frequency dependence on modulus and an absence of *G*′/*G*″ crossover at any temperature over the range explored (Figure 3D). The modulus for PDK-monovalent elastomers increased at all temperatures (Figure 3E) compared to the unfilled formulation (Figure 2E), for example, 250 kPa at 30 °C and 130 kPa at 150 °C in the carbon black filled formulation compared to 190 kPa at 30 °C and 40 kPa at 150 °C in the unfilled formulation. While we again observed a decrease in modulus with temperature, the magnitude of the decrease was reduced, particularly at higher temperatures. Thus, microstructural attributes of PDK networks, particularly the manner in which excess amine functionality is presented throughout, strongly influence the reinforcing characteristics of carbon black fillers, tying back to differences in structure and dynamic properties of the polyetheramine macromers at the carbon black interface.

These differences in the reinforcing effect also impact stress relaxation behavior for both PDK-multivalent and PDK-monovalent carbon-reinforced rubbers (Figure 3C,F). The relaxation kinetics for PDK-multivalent rubbers did not change appreciably with the inclusion of carbon black, providing further evidence that excess amine functionality does not appreciably interact with the filler and that diketoenamine bond exchange permitting relaxation is highly localized. Conversely, relaxation in PDK-monovalent rubbers was substantially slower with the inclusion of carbon black, requiring >10-fold longer to relax to e^–1^ compared to the unfilled sample. Thus, displaying amine functionality at sterically less hindered sites throughout the networks of PDK-monovalent elastomers permits their adsorption to filler surfaces, and the relaxation behavior tied to that adsorption reflects slower chain dynamics at the filler interface. When taken together, the results for unfilled and filled PDK elastomers validate our overarching hypothesis by illustrating that multivalency in the flexible amine macromer is key to the creation of cross-linked PDK elastomers and rubbers that resist long-range viscous flow yet retain capacity for short-range bond exchange to remain mechanically processable during thermoforming.

### PDK Elastomers with Multivalent Soft Segments Resist Creep

**Figure 4:**
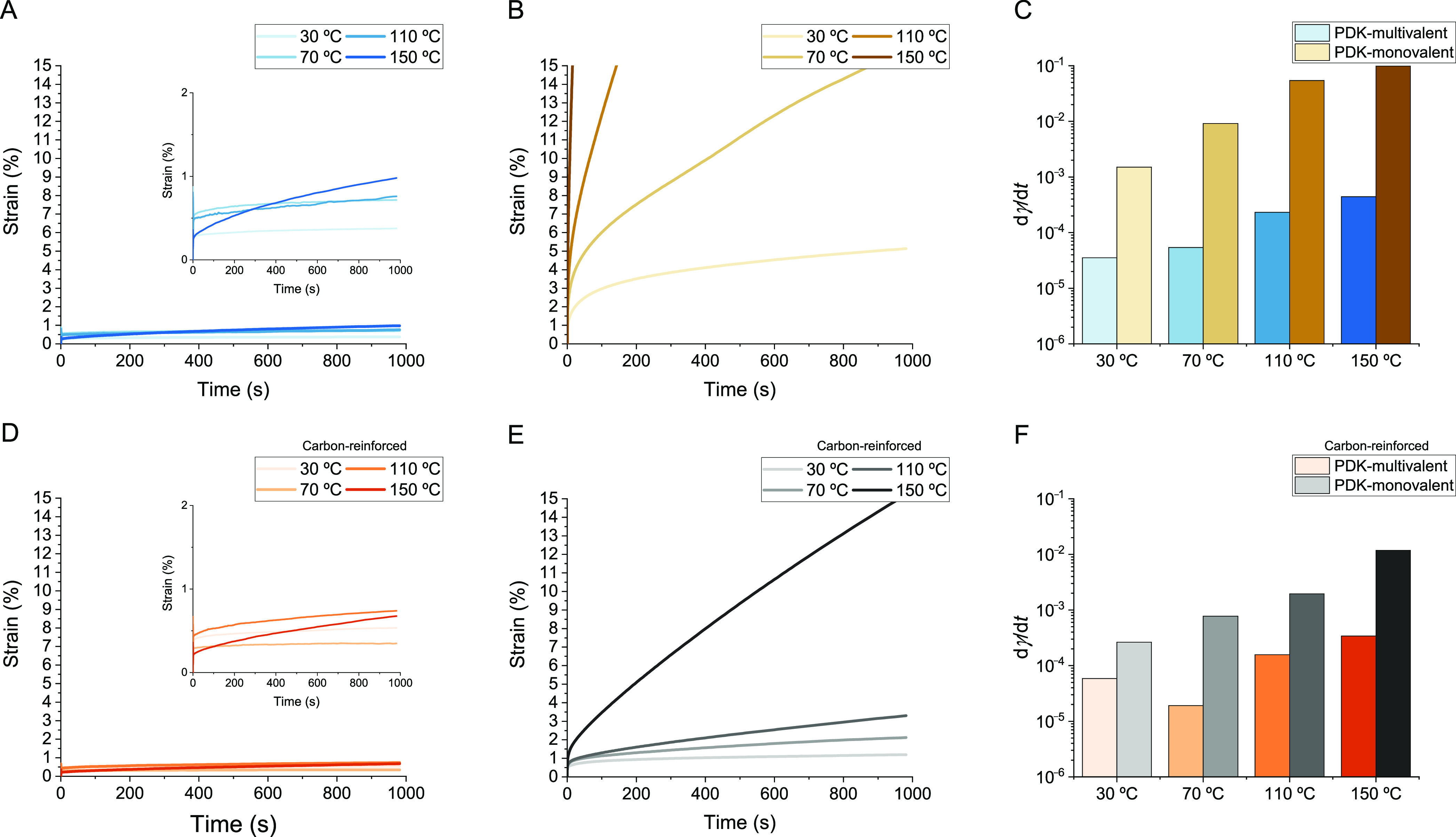
Creep and residual strain rates in unfilled and reinforced PDK elastomers. (A) PDK-multivalent elastomer creep, showing exceptional creep resistance at all temperatures. (B) PDK-monovalent elastomer creep, showing high susceptibility to creep at all temperatures. (C) Strain rate (dγ/d*t*) versus temperature for PDK-multivalent and PDK-monovalent elastomers. (D) PDK-multivalent carbon-reinforced (0.5 wt %) rubber creep, showing exceptional creep resistance at all temperatures. (E) PDK-monovalent carbon-reinforced (0.5 wt %) creep, showing improved creep resistance at all temperatures. (F) Strain rate (dγ/d*t*) versus temperature for PDK-multivalent and PDK-monovalent carbon-reinforced (0.5 wt %) elastomers. Strain rate was calculated from the last 200 s of the strain versus time data.

While dynamic covalent polymers are a promising platform for producing recyclable thermosets, bond exchange can lead to creep, which diminishes their use in applications that cannot tolerate material deformation under an applied load. We measured creep in both PDK-multivalent and PDK-monovalent elastomer networks under 1 kPa stress and observed remarkably low creep for PDK-multivalent, with no sample reaching greater than 1% strain up to 150 °C (Figure 4A). By contrast, PDK-monovalent flowed readily, due to high chain mobility, reaching >200% strain at 150 °C (Figure 4B). We calculated the residual strain rate from a linear fit of the last 200 s of the strain versus time data; the strain rate was up to 2 orders of magnitude lower for PDK-multivalent elastomers than for PDK-monovalent elastomers, which reflects an increase in network viscosity. Adding carbon black to PDK-multivalent elastomers produced a small decrease in creep relative to unfilled materials (Figure 4D), with all samples reaching a strain ≤0.7% up to 150 °C. This behavior continued to stand out, even when we added carbon black to PDK-monovalent elastomers, which reduced creep by up to 13-fold relative to unfilled PDK-monovalent—although it was clear that continuous deformation could not be avoided at elevated temperature (Figure 4E).

The residual strain rates for PDK-multivalent elastomers with and without carbon black were of comparable magnitudes (Figure 4C,F), and similar to the unfilled samples, PDK-multivalent elastomers had an approximately order of magnitude lower strain rate than PDK-monovalent elastomers when carbon black was added. Given that the modulus of PDK-multivalent elastomers had been observed to increase with the addition of carbon black, there is a clear effect in reducing chain mobility. However, carbon black had little effect on the rate of stress relaxation and the magnitude of creep in PDK-multivalent elastomers. Furthermore, PDK-multivalent elastomers with and without carbon black had little temperature dependence on creep below 150 °C. A plausible explanation is that the number of bonds that must simultaneously break and re-form to allow long-range chain motion is sufficiently high in the unfilled material; carbon black simply increases the entropy penalty of deforming individual chain segments, resulting in an increase in modulus. We further conclude that introducing exchangeable bonds into linear segments in PDK-monovalent elastomers leads to a reduction in cross-link density at elevated temperature and produces undesirably high creep. From our earlier analysis, this reduction is due to reduced noncovalent entanglements enabled by bond exchange and network reorganization. Indeed, recent studies have demonstrated that increasing primary chain length in associative networks can reduce viscoelastic flow through maintaining molecular entanglements^43^ and that reducing the number of exchangeable bonds in the linear segment is critical to realizing this behavior.

### PDK Elastomers Exhibit High Thermal Stability

Encouraged by the mechanical stability of PDK-multivalent at high temperature, we further investigated its thermal stability to evaluate the potential for high service temperature applications without deleterious thermal degradation. We measured <1% mass loss in both PDK-multivalent (Figure S6) and PDK-monovalent (Figure S7) with or without carbon black after 10 000 s at 150 °C by TGA, verifying excellent thermal stability at the highest temperature in our rheological experiments. The decomposition temperature at 50% mass loss for PDK-multivalent was 419 °C without carbon black and 421 °C with carbon black (Figure S8), which was slightly higher than but comparable to PDK-monovalent (417 and 418 °C respectively, Figure S9), suggesting that the thermal stability arises from the network chemistry and not necessarily the cross-linking structure. To put these results in the context of thermal performance for conventional polymer formulations, we compared the decomposition temperatures of PDK-multivalent and PDK-monovalent at 50% weight loss to published data on polyurethanes that contained pTHF (*M*_n_ = 2000 g mol^–1^) as a soft segment.^44−47^ We assumed that 100% of the polyol content for the published formulations could be derived from biorenewable sources, and we plotted decomposition temperature against the mass fraction of biorenewable content (Figure S10). Our formulation shows a clear improvement in thermal stability with a relatively high biorenewable content, demonstrating the feasibility for deploying PDK-based elastomers in demanding environmental conditions.

### PDK Chemical Depolymerization Requires Heteroatom Proximity to the Diketoenamine Bond

**Figure 5:**
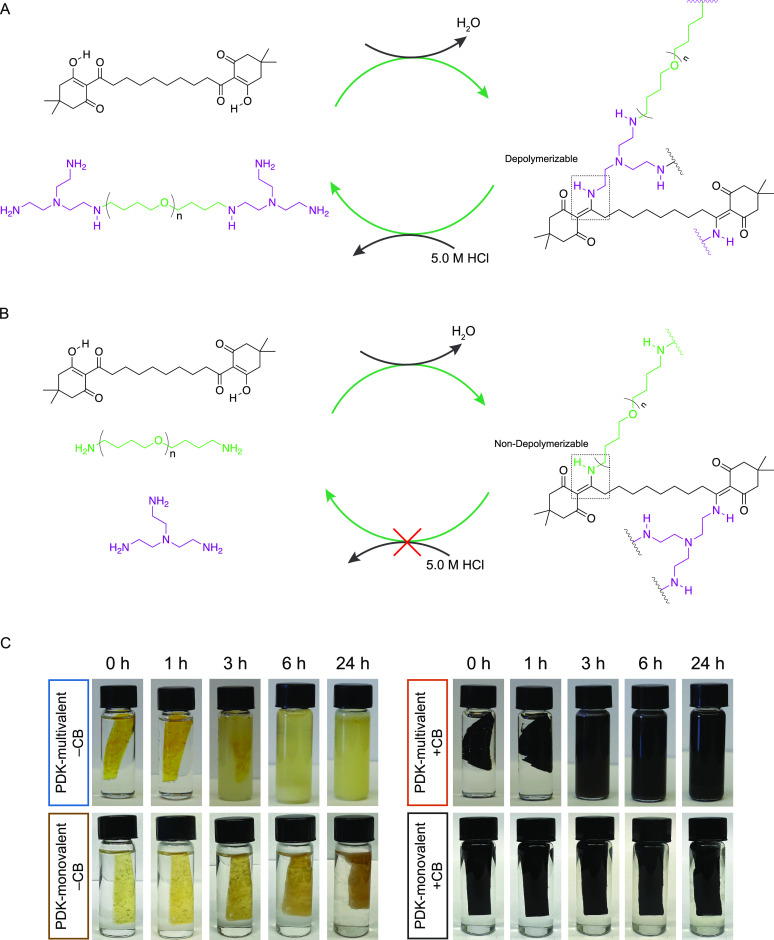
Chemical depolymerization requires heteroatom proximity to the diketoenamine bond. (A) TK-10 and pTHF-bis-TREN form cross-linked elastomers through a condensation polymerization, and they are depolymerized back to starting materials in the presence of aqueous HCl. (B) TK-10, pTHF-diamine, and TREN form cross-linked elastomers through a similar mechanism, but the diketoenamine bond formed between TK-10 and pTHF-diamine is nondepolymerizable in aqueous HCl. (C) Chemical depolymerization of PDK-multivalent and PDK-monovalent elastomers with and without carbon black. Depolymerization experiments were performed in 5.0 M HCl at ambient temperature.

Given the influence of amine macromer valency on the structure and dynamic properties of associated elastomers and carbon-reinforced rubbers, we probed whether these architectural attributes were in any way influential in their deconstruction behavior. We hypothesized that the higher cross-linking density of PDK-multivalent elastomers might slow their deconstruction to starting materials in strong acid. We further hypothesized that achieving high material efficiency in starting material recovery for PDK-monovalent elastomers might be compromised when the amine macromers are comprised of a mixture of compounds, in this case pTHF-diamine and TREN as the cross-linker. To evaluate the effects of PDK elastomer architecture on depolymerization rates, we incubated thermoformed samples in 5.0 M hydrochloric acid at ambient temperature for 24 h (Figure 5A,B). To our surprise (and invalidating these initial hypotheses), after only 6 h, PDK-multivalent samples both with and without carbon black had depolymerized to starting material, whereas PDK-monovalent samples swelled and softened, but remained intact after 24 h (Figure 5C). We recovered TK-10 and pTHF-bis-TREN components from depolymerized PDK-multivalent elastomers in 90% yield. Both were identical to pristine starting materials by NMR spectroscopy and MALDI-ToF mass spectrometry (Figure S11). To understand the origins of this behavior, we recognized that the end-group structure of pTHF diamine and TREN are inequivalent. Because TREN is known to promote facile PDK deconstruction, and does so expediently for PDK-multivalent elastomers when TREN end-caps pTHF-bis-TREN cross-linkers, we hypothesized that the proximity of the tertiary amine to the diketoenamine bond may play an important role in acidolysis and therefore PDK depolymerization rates.

**Figure 6:**
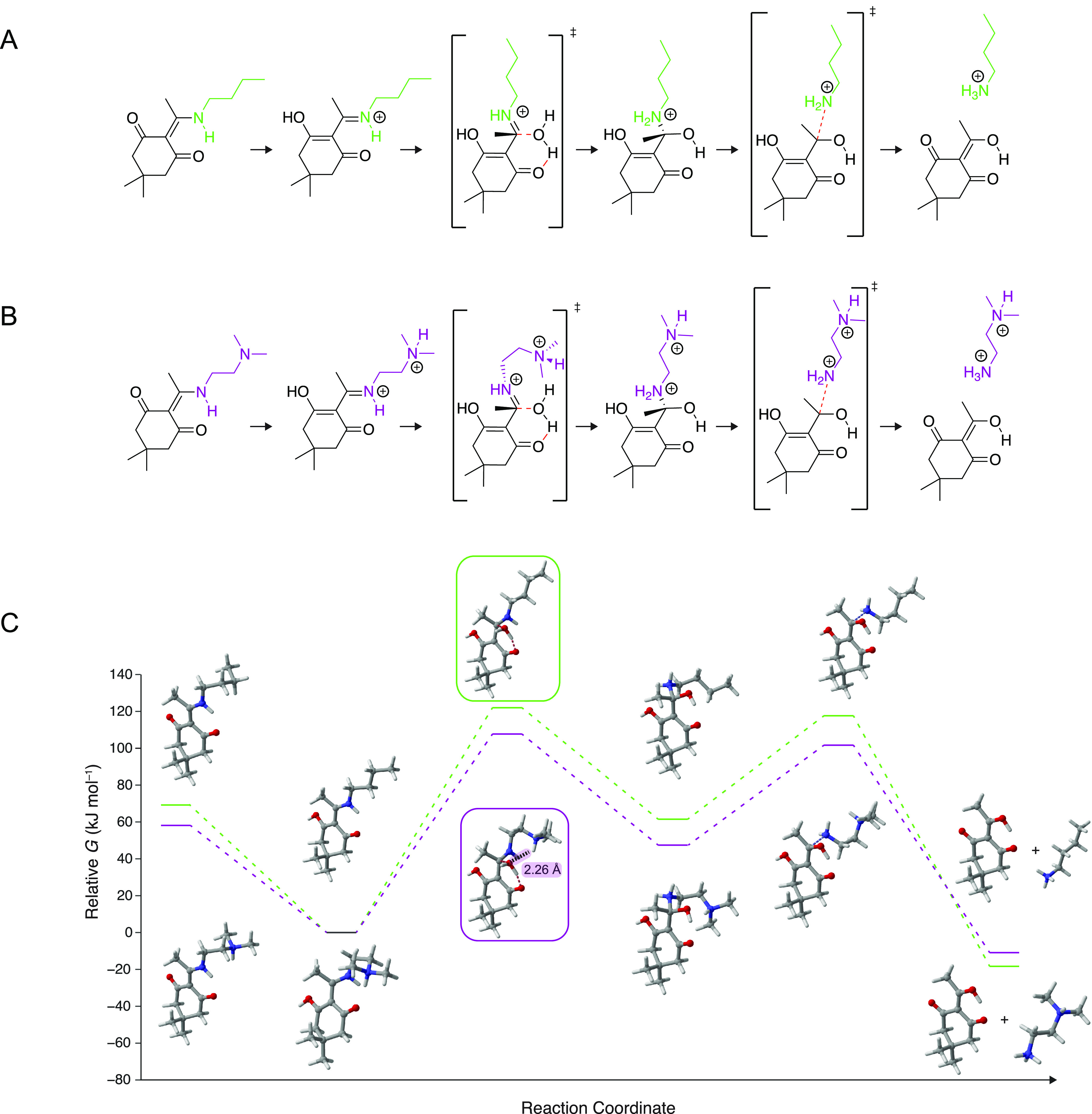
Computational reaction coordinates for acid-catalyzed diketoenamine hydrolysis. (A) Chemical structures of small-molecule analogues of PDK-monovalent using acyl dimedone and *n*-butylamine. (B) Chemical structures of small-molecule analogues of PDK-multivalent using acyl dimedone and *N*,*N*-dimethylaminoethylamine. (C) Computational reaction coordinate of PDK-monovalent (top series in green) and PDK-multivalent (bottom series in purple).

To test this revised hypothesis, we sought a mechanistic understanding of how heteroatom proximity affects depolymerization energetics. We carried out a computational simulation of acid-catalyzed diketoenamine hydrolysis using small-molecule surrogates for pTHF-bis-TREN and pTHF-diamine: specifically, diketoenamines featuring either a butyl group or an *N*,*N*-dimethylaminoethyl group (Figure 6). The acidolysis of both diketoenamines is exergonic and is in fact more favorable for the pTHF-diamine surrogate than the pTHF-bis-TREN surrogate. However, the reaction kinetics ultimately explain why pTHF-bis-TREN is depolymerizable while pTHF-diamine is not. In the rate-limiting step, water adds to a protonated iminium intermediate along the reaction coordinate.^28,48^ Here, we found that the corresponding transition state for the butyl-functionalized diketoenamine has a standard free energy of activation approximately 13 kJ mol^–1^ greater than that for the *N*,*N*-dimethylaminoethyl-functionalized diketoenamine. This difference in free energy of activation is due to the fact that the tertiary amine of the *N*,*N*-dimethylaminoethyl group stabilizes the transition state via a strong hydrogen bond (2.26 Å) with the incoming water, and thus decreases the free energy of activation for the *N*,*N*-dimethylaminoethyl-functionalized diketoenamine. Thus, the multivalent pTHF-bis-TREN end-group structure, in addition to providing for useful and advantaged PDK properties as elastomers and rubbers, is also essential for ensuring complete and rapid PDK depolymerization to triketone and amine starting materials at ambient temperature in strong acid.

## Conclusions

We find that PDK elastomers address ongoing challenges in the creation of highly recyclable yet mechanically stable rubbers that remain thermoformable, due to their dynamic covalent cross-links. Elastomers prepared from commercially available pTHF-diamine soft segments contain exchangeable bonds that permit remolding, but their inability to undergo chemical recycling led us to discover that heteroatom proximity to the diketoenamine bond is required to enable depolymerization. Covalently attaching TREN moieties to the soft segment restored the ability to undergo monomer-to-monomer chemical recycling, and it also produced unexpected creep resistance that could not be achieved with the analogous PDK-monovalent formulation. This study advances fundamental insights into the macromolecular design of high-performance cross-linked elastomers that are amenable to circular manufacturing. We anticipate leveraging this platform in the future, both to broaden the range of properties exhibited and to enable chemical recycling of more complex products featuring circular PDK elastomers alongside other materials.

## Supporting Information Available

Materials, methods, computational parameters, ^1^H NMR of all compounds, MALDI characterization, FTIR spectroscopy, DSC, TGA, thermal property characterization (PDF)
Transparent Peer Review report available (PDF)
