# Supplementary material for: Circular Polydiketoenamine Elastomers with Exceptional Creep Resistance via Multivalent Cross-Linker Design
Source: ACS Cent Sci. 2023 Nov 17;10(1):54–64. doi: 10.1021/acscentsci.3c01096 (PMC10823519; doi:10.1021/acscentsci.3c01096)
Supplement: Supplementary file 1 — oc3c01096_si_001.pdf [file oc3c01096_si_001.pdf]

Supplemental Information

Circular Polydiketoenamine Elastomers with Exceptional Creep Resistance via Multivalent Crosslinker Design

Eric A. Dailing, Pawan Khanal, Alexander R. Epstein, Jeremy Demarteau, Kristin A. Persson, Brett A. Helms*

Eric A. Dailing
Molecular Foundry
Lawrence Berkeley National Laboratory
1 Cyclotron Road, Berkeley CA, 94270 USA

Pawan Khanal
Materials Sciences and Engineering
University of California, Berkeley
Berkeley, CA 94720 USA

Alexander R. Epstein
Materials Sciences and Engineering
University of California, Berkeley
Berkeley, CA 94720 USA

Jeremy Demarteau
Molecular Foundry
Lawrence Berkeley National Laboratory
1 Cyclotron Road, Berkeley CA, 94270 USA

Kristin A. Persson
Molecular Foundry
Lawrence Berkeley National Laboratory
1 Cyclotron Road, Berkeley CA, 94270 USA
and
Materials Sciences Division
Lawrence Berkeley National Laboratory
1 Cyclotron Road, Berkeley CA, 94270 USA
and
Materials Sciences and Engineering
University of California, Berkeley
Berkeley, CA 94720 USA

Brett A. Helms*
Molecular Foundry
Lawrence Berkeley National Laboratory
1 Cyclotron Road, Berkeley CA, 94270 USA
and

Materials Sciences Division
Lawrence Berkeley National Laboratory
1 Cyclotron Road, Berkeley CA, 94270 USA
Email: bahelms@lbl.gov

Materials

Poly(tetrahydrofuran) (pTHF) ($M_n \approx 2{,}000$ g mol^{-1}), methanesulfonyl chloride (>99%), triethylamine (>99%), *tris*(2-aminoethyl)amine (TREN, 96%), 5,5-dimethyl-1,3-cyclohexanedione (dimedone, 95%), sebacic acid (99%), *N*,*N′*-dicyclohexylcarbodiimide (DCC, 99%), 4-(dimethylamino)pyridine (DMAP, >99%), sodium hydroxide (>97%), magnesium sulfate (anhydrous, >99.5%), Amberlyst A26 hydroxide form (basic) resin, and sodium trifluoroacetate (99%) were received from Sigma-Aldrich. Dichloromethane (>99.9%), chloroform (>99.8%), tetrahydrofuran (>99%), methanol (>99%) and concentrated hydrochloric acid were received from VWR. Carbon black (Super P conductive, >99%) was received from Alfa Aesar. Chloroform-*d* (99.8% *D*) was received from Cambridge Isotope Laboratories. Dithranol (99%) was received from MP Biomedicals. Poly(tetrahydrofuran) diamine (pTHF-diamine) was received from Huntsman Chemical, and we calculated M_n = 1,535 g mol^{-1} and Đ = 1.21 via MALDI.

Instrumentation

Nuclear Magnetic Resonance Spectroscopy (NMR). ^{1}H NMR spectra was recorded on a Bruker Avance II at 500 MHz. Chemical shifts are reported in δ (ppm) relative to $CDCl_3$ at 7.26 ppm.

Matrix-Assisted Laser Desorption/Ionization Time-of-Flight Mass Spectrometry (MALDI-ToF). MALDI mass spectra were recorded on a Bruker rapifleX spectrometer in positive reflector mode. A solution containing analyte (1 mg mL^{-1}) and dithranol (10 mg mL^{-1}) was prepared in THF, and 1 μL of this mixture was applied to a stainless-steel target plate and allowed to dry completely before analysis.

Fourier-Transform Infrared Spectroscopy (FT-IR). FT-IR spectra were recorded on a Thermo-Fisher Nicolet iS50 spectrometer in Attenuated Total Reflectance (ATR) mode.

Rheological Analysis. Amplitude sweep, frequency sweep, stress relaxation, and creep measurements were performed on a TA DHR-2 rheometer. Elastomer samples were cut into 8 mm discs with a biopsy punch and loaded onto a rheometer between 8-mm stainless steel parallel plates.

Differential Scanning Calorimetry (DSC). DSC measurements were performed on a TA Q200 from –80 to 100 ºC with a temperature ramp of 10 ºC min^{-1}. Data is reported for the second heating cycle for each sample.

Thermogravimetric Analysis (TGA). TGA measurements were performed on a TA 5500 from 150 to 800 ºC with a 120 min isothermal hold at 150 ºC and a 10 ºC min^{-1} temperature ramp. Isothermal measurements were performed at 150 ºC for 10,000 s. All measurements were performed under nitrogen atmosphere.

Tensile testing. Tensile measurements were performed on an Instron 68TM-5 with 1 kN load cell at ambient temperature. Dog bone samples were prepared with width 4.5 mm, thickness 1 mm, and gauge length 25 mm. Samples were strained to failure at a tensile rate of 50 mm min^{-1}.

Methods

Synthesis of Ditopic Triketone Monomer, TK-10.

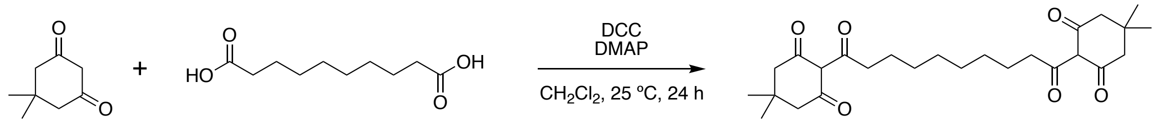

The ditopic triketone monomer, TK-10, was synthesized as previously described.[1] Briefly, a round bottom flask was charged with dimedone (20.0 g, 0.143 mol), DMAP (24.9 g, 0.204 mol), and dichloromethane (250 mL). A solution of DCC (33.7 g, 0.163 mol) in dichloromethane (80 mL) was added dropwise at room temperature, and the reaction was allowed to proceed for 18 h. The resulting solution was filtered to remove dicyclohexylurea, and the organic phase was washed three times with 10% HCl. The product was concentrated under reduced pressure to obtain a yellow-orange solid. The crude product was dissolved in 1.0 M NaOH, washed three times with dichloromethane, and acidified with 1.0 M HCl to precipitate an off-white solid. The product was collected by filtration and dried under reduced pressure. ^{1}H NMR analysis of the product was consistent with the previous report (see below).[1]

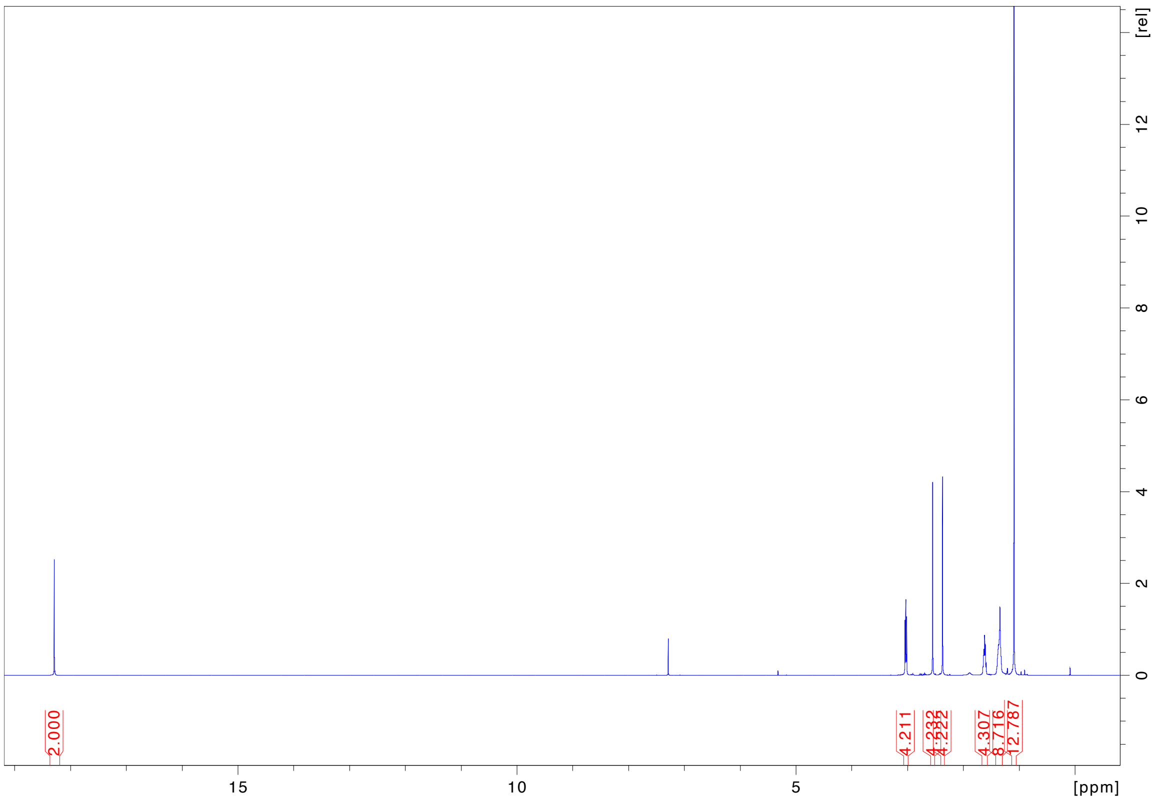

^{1}H NMR spectrum of **TK-10**

Synthesis of pTHF-*bis*-mesylate.

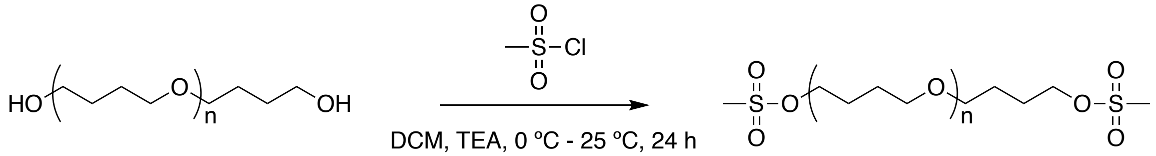

pTHF (30 g, 0.015 mol) was dissolved in dichloromethane (600 mL) in a round bottom flask with stirring. Triethylamine (10.6 g, 0.105 mol) was added, and the flask was transferred to an ice bath. Methanesulfonyl chloride (6.01 g, 0.053 mol) was dissolved in dichloromethane (20 mL) and added dropwise under nitrogen atmosphere. The reaction was stirred at 0 ºC for 1 h, after which the ice bath was removed, and the reaction was stirred for 18 h at room temperature. The reaction mixture was concentrated to 200 mL DCM, combined with DI water (100 mL) and stirred for 30 min at room temperature. The solution was transferred to a separatory funnel and the organic phase was washed 3x with DI water. The organic phase was then dried over magnesium sulfate and the solvent was removed under reduced pressure to yield a waxy orange solid (25.6 g, 79%). ^{1}H NMR (500 MHz, $CDCl_3$, 25 ºC, TMS): δ 4.25 (t, *J*=6.51 Hz, 4H; CH_2-CH_2-O-SO_2-CH_3), 3.49-3.33 (m, 142H; (CH_2-CH_2-O)$_n$), 2.99 (s, 6H; CH_3), 1.70-1.52 (m, 143H; (CH_2-CH_2-O)$_n$).

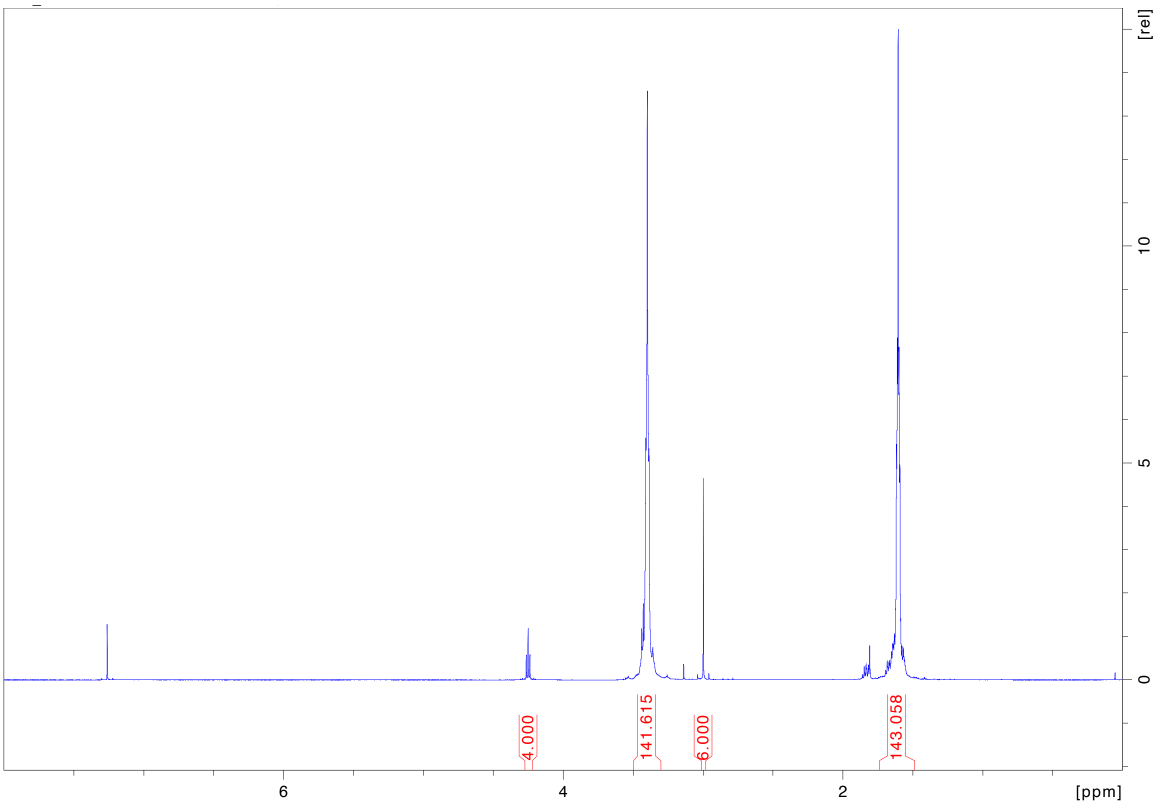

^{1}H NMR spectrum of **pTHF-*bis*-mesylate**.

Synthesis of pTHF-*bis*-TREN

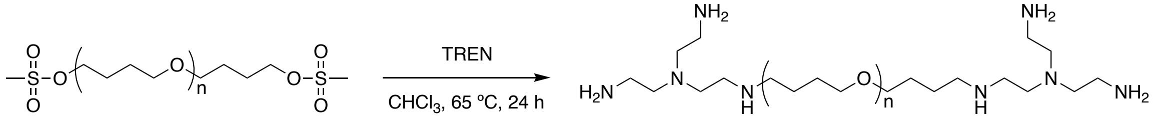

pTHF-*bis*-mesylate (20 g, 0.01 mol) was dissolved in chloroform (120 mL). A round bottom flask was charged with TREN (35.2 g, 0.24 mol) and heated to 65 ºC in an oil bath. The pTHF-*bis*-mesylate solution was added dropwise at 0.2 mL min^{-1} and stirred for an additional 12 h at 65 ºC. The crude reaction mixture was diluted with dichloromethane and filtered to remove TREN-sulfonate salts, and the soluble fraction was stirred for 18 h with Amberlyst A26 OH ion exchange resin. The resin was removed by filtration and the organic solvents were subsequently removed under reduced pressure to yield a heterogeneous yellow mixture. DI water (200 mL) was added and the mixture was divided into 50-mL centrifuge tubes and centrifuged at 10,000 rpm for 5 min. The recovered white solids were washed 2x with DI water and centrifuged after each wash step. The solids were collected in methanol and dried under reduced pressure to yield a waxy white solid (9.3 g, 47%). (500 MHz, $CDCl_3$, 25 ºC, TMS): δ 3.50-3.30 (s, 102H; $(CH_2\text{-}CH_2\text{-}O)_n$), 2.80-2.48 (m, 24H; N-$CH_2$-$CH_2$-$NH_2$), 1.76-1.47; (s, 115H; $(CH_2\text{-}CH_2\text{-}O)_n$). M_n = 2,203 g mol^{-1}, Đ = 1.07 (MALDI).

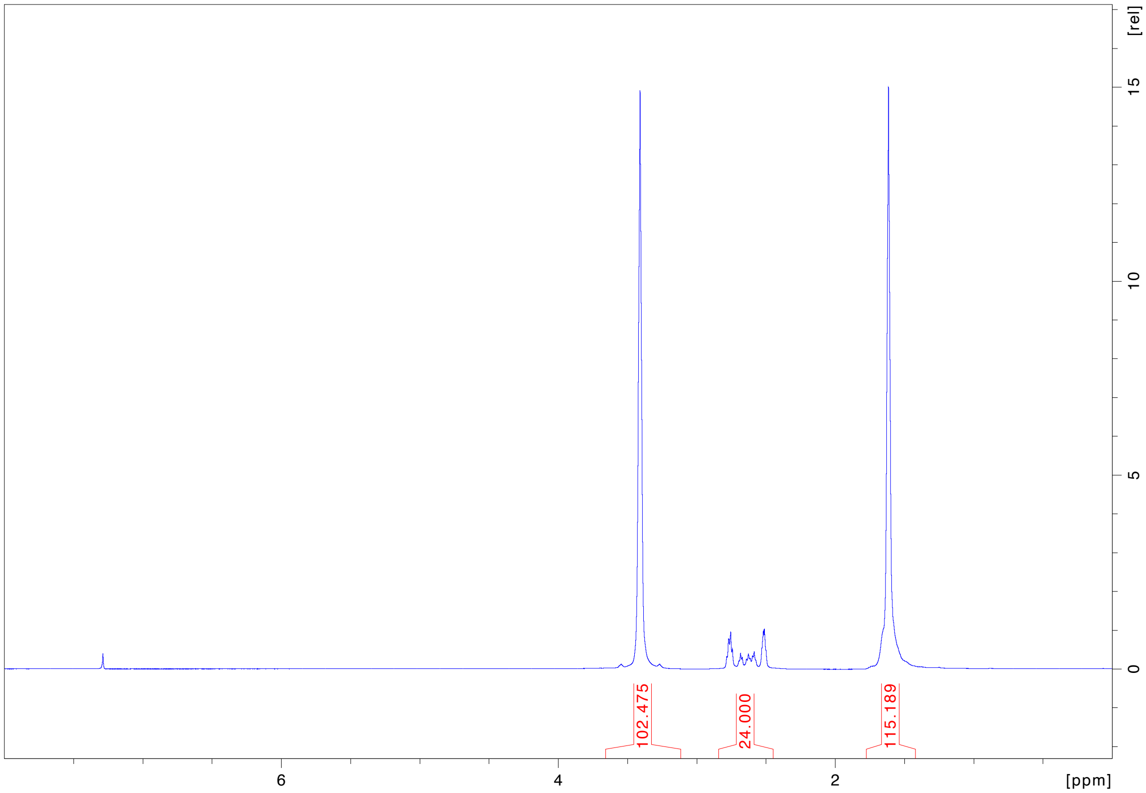

^{1}H NMR spectrum of **pTHF-*bis*-TREN**.

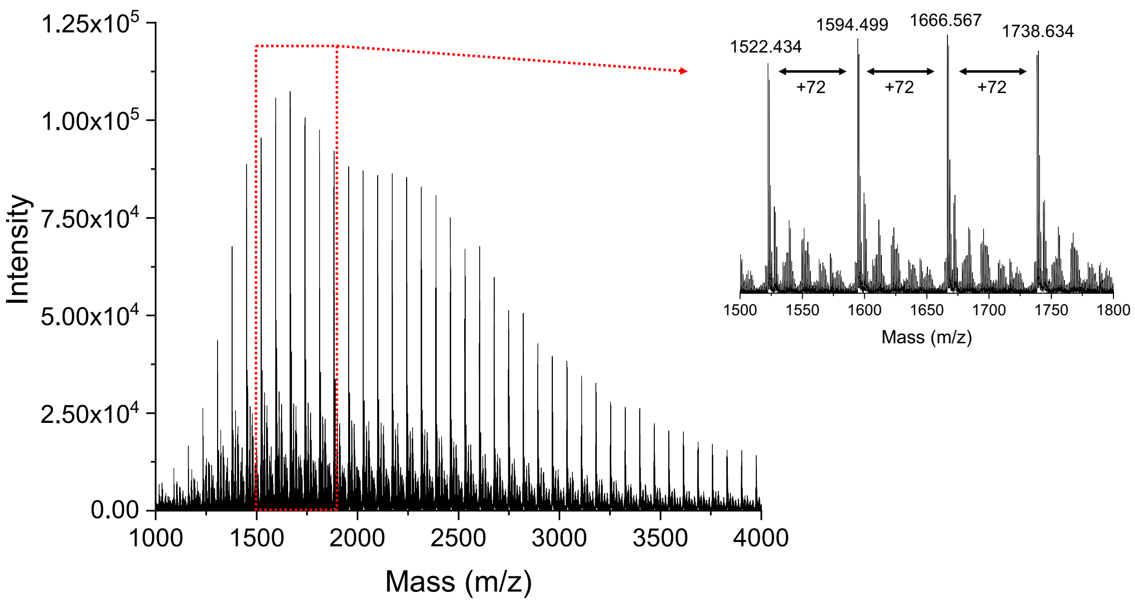

MALDI-ToF mass spectrum of **pTHF-*bis*-TREN**.

Synthesis of PDK-multivalent Elastomers.

In a typical synthesis, pTHF-*bis*-TREN (1.0 g, 0.45 mmol, which gives 1.8 mmol amines) was dissolved in THF (1.0 mL) in a glass vial and heated to 60 ºC. TK-10 (0.31 g, 0.69 mmol, which gives 1.38 mmol triketones) was separately dissolved in THF (0.31 mL) in a glass vial and heated to 60 ºC. The TK-10 solution was rapidly added to the pTHF-*bis*-TREN solution and the mixture was stirred with a metal spatula. After approximately 30 s, a solid gel was obtained. The heat was increased to 75 ºC and the gel was dried under vacuum to remove residual THF and water generated from the diketoenamine condensation. For samples containing carbon black, a solution of 0.5% w/v carbon black in THF was prepared by sonication, and combined with pTHF-*bis*-TREN and TK-10 as described. Elastomer samples were pressed in Teflon molds using a Stahls' Hotronix heat press at 150 ºC and 60 psi for 5 min.

Synthesis of PDK-monovalent Elastomers.

pTHF-diamine (4.0 g, 2.6 mmol, which gives 5.2 mmol amines) and TK-10 (1.25 g, 2.80 mmol, which gives 5.6 mmol triketones) were combined in a glass vial and heated to 110 ºC with stirring for 30 min until the mixture became homogeneous and evolution of bubbles ceased. The melt was cooled to 60 ºC, and TREN (0.1 g, 0.69 mmol, which gives 2.07 mmol amines) was added rapidly. The mixture was stirred with a metal spatula to obtain a viscous paste. The mixture was dried under vacuum at 70 ºC to remove residual water. For samples containing carbon black, a solution of 0.5% w/v carbon black in THF was prepared by sonication, and combined with pTHF-diamine and TK-10 as described. Elastomer samples were pressed in Teflon molds using a Stahls' Hotronix heat press at 150 ºC and 60 psi for 5 min.

Gel fraction measurements.

Elastomer samples were pressed as described above, cut into 8 mm x 1 mm discs with a biopsy punch, and weighed. Samples were incubated in 4 mL chloroform at room temperature for 48 h. Chloroform was exchanged 8 times over this period. Samples were then dried to constant mass under vacuum and the final mass was recorded. The gel fraction for PDK-monovalent was 94.1% and the gel fraction of PDK-multivalent was 96.3%.

Bulk density measurements.

The bulk density of the crosslinked elastomers was measured using the density bottle method. The empty mass of an oven-dried density bottle with capillary stopper was recorded (m_1) and loaded with a pressed elastomer sample that was cut into a 8 mm x 1 mm disc. Sample mass was recorded (m_2). The bottle was then filled with DI water that was equilibrated at 20 ºC and the total mass was recorded (m_3). Finally, the sample was removed and the bottle was refilled with DI water and the mass was recorded (m_4). The specific gravity of each sample was determined from the equation $g = \frac{m_2 - m_1}{(m_2 - m_1) - (m_3 - m_4)}$, and the result was converted to density using the measured density of DI water at 20 ºC (0.998 g mL^{-1}). The calculated density for PDK-monovalent was 1.15 g mL^{-1} and the density for PDK-multivalent was 1.11 g mL^{-1}.

Depolymerization of PDK elastomers.
Elastomer samples were incubated in a solution of 5.0 M HCl at room temperature with stirring. Samples were centrifuged to pellet the precipitated TK-10, and the supernatant containing dissolved pTHF-*bis*-TREN□HCl was reserved. Solid TK-10 was washed with DI water and dried under vacuum. The aqueous solution containing pTHF-*bis*-TREN□HCl was stirred with Amberlyst A26 OH resin, and water was removed by distillation to yield pTHF-*bis*-TREN as the free base.

Computational Methods.

All hybrid-DFT calculations were performed using Gaussian16[2]. Input files were prepared and output files were parsed using Pymatgen[3]. All free energies were calculated at the M062-X/6-311+G(d,p)//SMD level of theory using the Quasi-Rigid Rotor Harmonic Oscillator (Quasi-RRHO) method for calculating the vibrational entropy[4–6]. To find the lowest-energy conformers contributing to the free energy of activation, we performed a conformer search on the ground state and transition state structures according to the procedure depicted in the following flow chart. For transition states, the optimization at the B97D[7] level of theory was done with a fixed explicit water molecule involved in the transition state. Uniqueness was defined as structures with electronic energies greater than 0.1 kJ mol^{-1} apart and RMSDs greater than 0.1 Å apart.

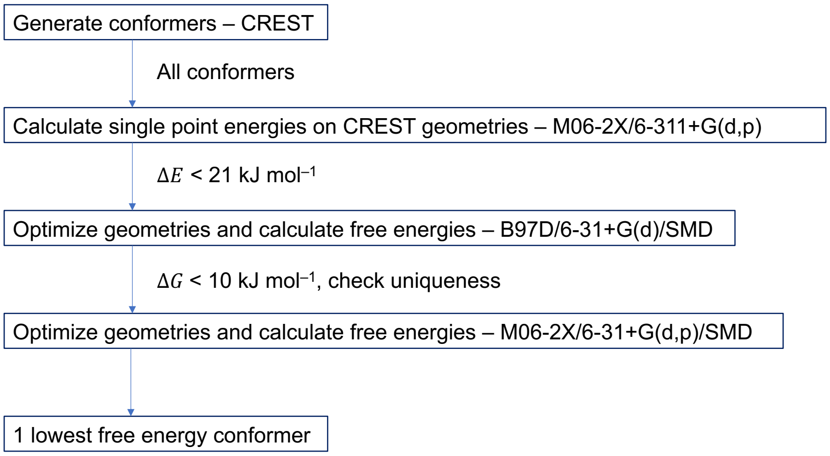

We note that in the conformer search for the ground state of the the *N*,*N*-dimethylaminoethyl-functionalized diketoenamine, the lowest-energy conformer does not match the configuration of the butyl-functionalized diketoenamine – the iminium does not form a hydrogen bond with the ketone. However, that conformation is similar in free energy and its use would not change the qualitative trend that the *N*,*N*-dimethylaminoethyl-functionalized diketoenamine has a significantly (> 10 kJ mol^{-1}) lower free energy of activation than the butyl-functionalized diketoenamine.

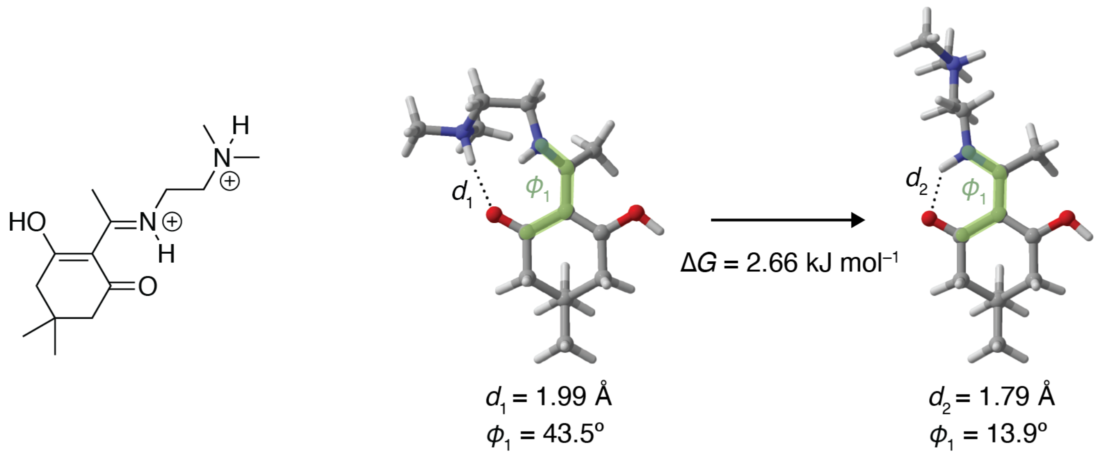

For the *N*,*N*-dimethylaminoethyl-functionalized diketoenamine, all other structures along the reaction coordinate were taken from previous work[1]. For the diketoenamines featuring a butyl group, all other structures along the reaction coordinate were found by geometry optimization following substitution of the *N*,*N*-dimethylaminoethyl group, *i.e.* without an additional conformer search.

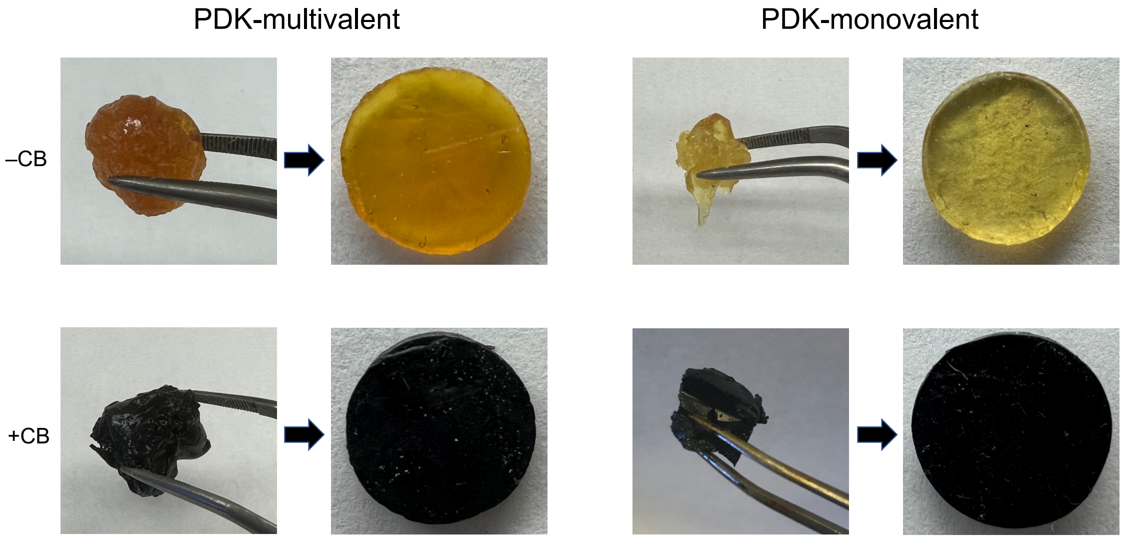

Figure S1. Photographs of elastomer samples before and after reprocessing in a circular Teflon mold at 150 ºC and 60 psi for 300 s.

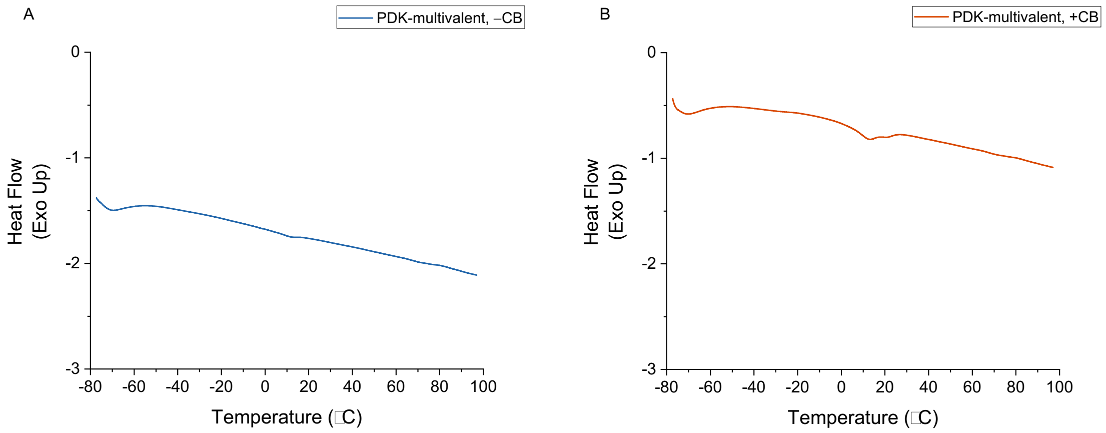

Figure S2. DSC traces of PDK-multivalent elastomers: **A,** without carbon black, and **B,** with 0.5 wt% carbon black.

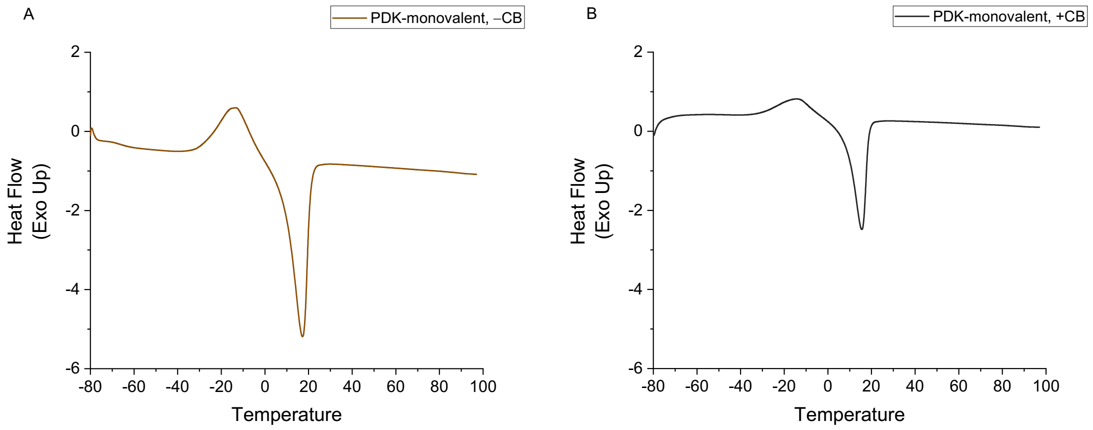

Figure S3. DSC traces of PDK-monovalent elastomers: **A,** without carbon black, and **B,** with 0.5 wt% carbon black.

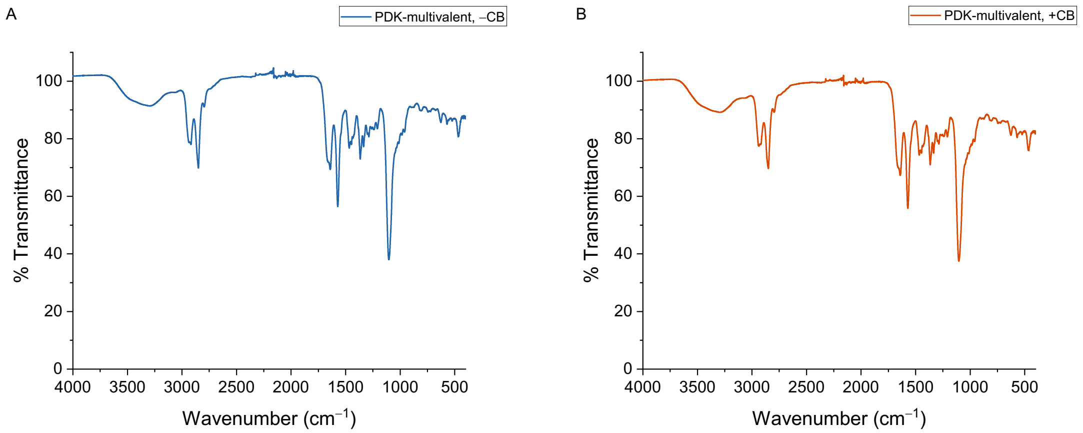

Figure S4. ATR-FTIR spectra of PDK-multivalent elastomers: **A,** without carbon black, and **B,** with 0.5 wt% carbon black.

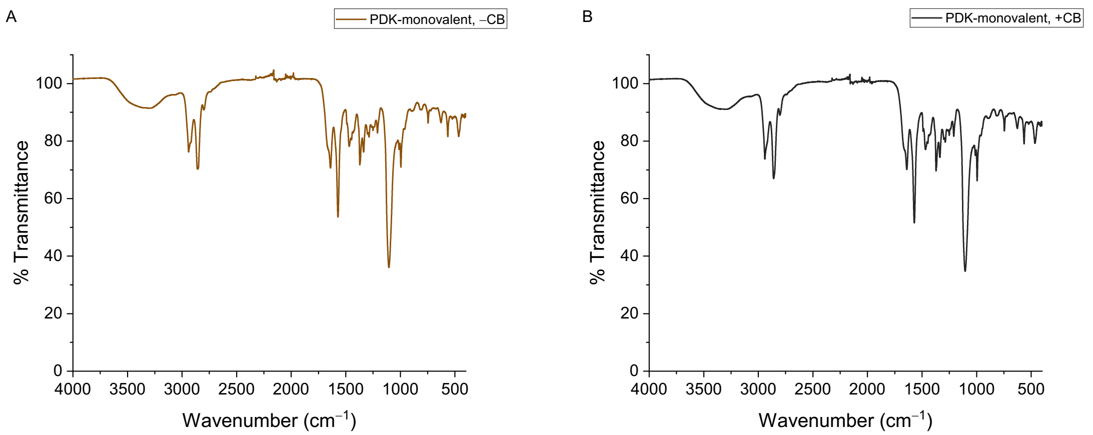

Figure S5. ATR-FTIR spectra of PDK-monovalent elastomers: **A,** without carbon black, and **B,** with 0.5 wt% carbon black.

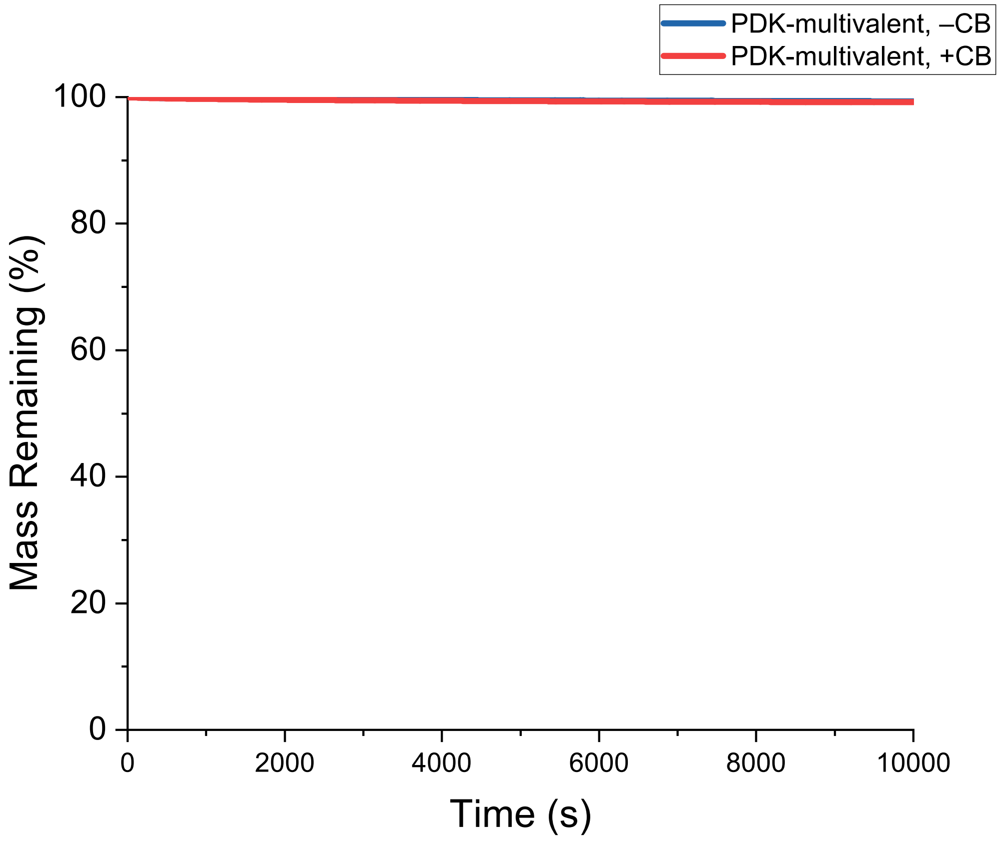

Figure S6. TGA isothermal runs for PDK-multivalent elastomers with or without 0.5 wt% carbon black. Runs were performed under nitrogen at 150 ºC for 10,000 s.

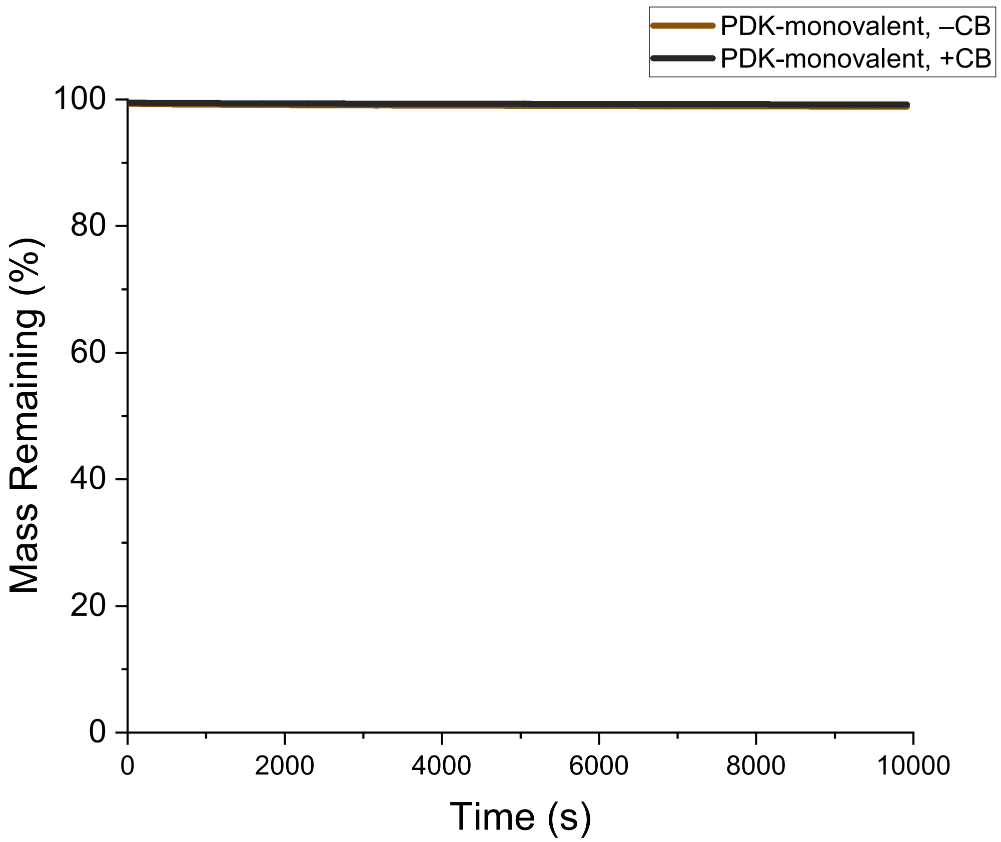

Figure S7. TGA isothermal runs for PDK-monovalent elastomers with or without 0.5 wt% carbon black. Runs were performed under nitrogen at 150 ºC for 10,000 s.

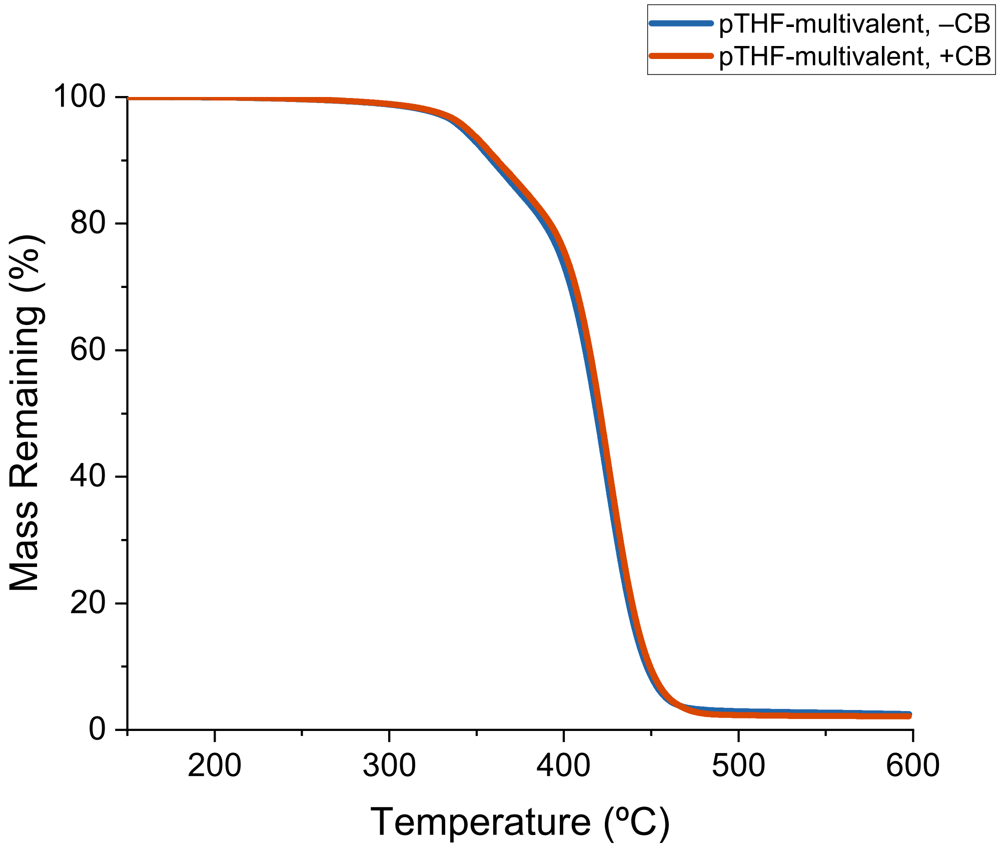

Figure S8. TGA temperature ramps for PDK-multivalent elastomers with or without 0.5 wt% carbon black. Runs were performed under nitrogen from 150 ºC to 600 ºC.

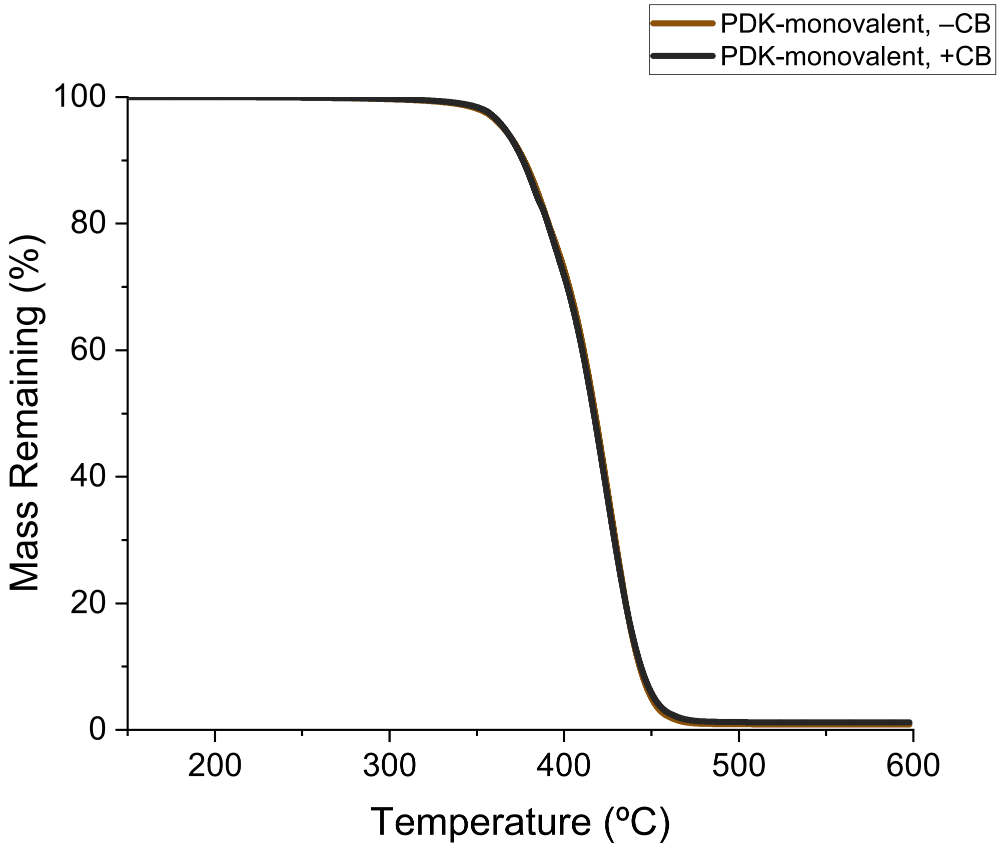

Figure S9. TGA temperature ramps for PDK-monovalent elastomers with or without 0.5 wt% carbon black. Runs were performed under nitrogen from 150 ºC to 600 ºC.

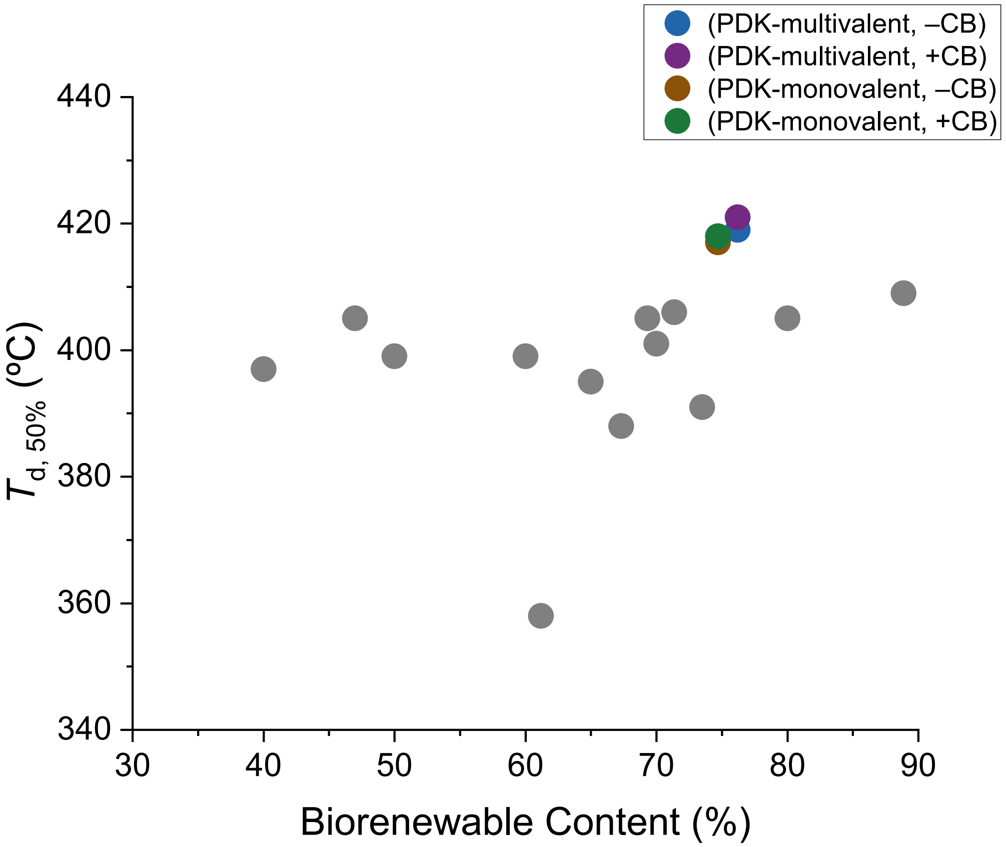

Figure S10. Thermal stability as a function of the mass fraction of biorenewable content for PDK elastomers with or without 0.5 wt% carbon black. Grey data points are extracted from published data[8–11] for temperature at 50% weight loss.

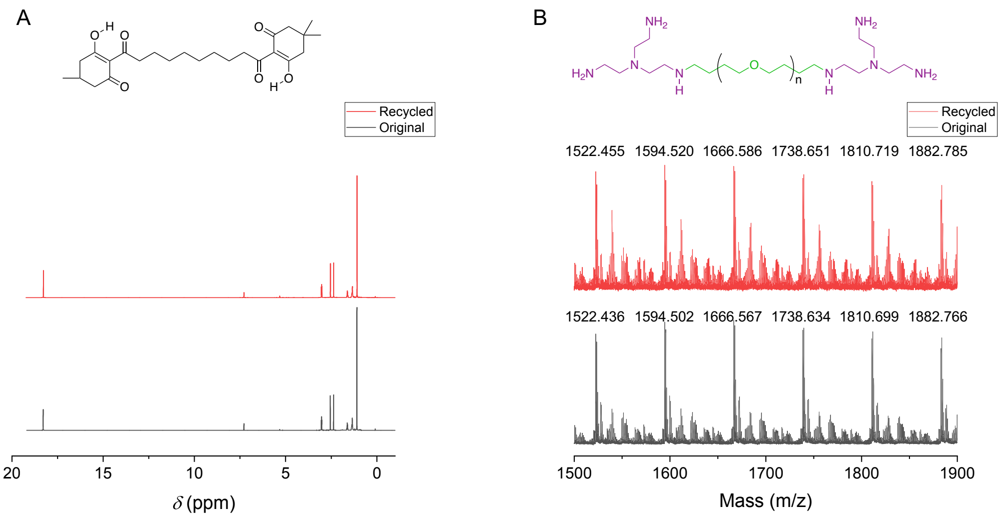

Figure S11. a, ^{1}H NMR spectra of recycled and pristine TK-10. **b,** MALDI mass spectra of recycled and pristine pTHF-*bis*-TREN.

Supplemental References

(1) Christensen, P. R.; Scheuermann, A. M.; Loeffler, K. E.; Helms, B. A. Closed-Loop Recycling of Plastics Enabled by Dynamic Covalent Diketoenamine Bonds. *Nat. Chem.* **2019**, *11* (5), 442–448. https://doi.org/10.1038/s41557-019-0249-2.

(2) Frisch, M. J.; Trucks, G. W.; Schlegel, H. B.; Scuseria, G. E.; Robb, M. a.; Cheeseman, J. R.; Scalmani, G.; Barone, V.; Petersson, G. a.; Nakatsuji, H.; et al. G16_C01. 2016, p Gaussian 16, Revision A.03, Gaussian, Inc., Wallin.

(3) Ping, S.; Davidson, W.; Jain, A.; Hautier, G.; Kocher, M.; Cholia, S.; Gunter, D.; Chevrier, V. L.; Persson, K. A.; Ceder, G. Python Materials Genomics (Pymatgen): A Robust , Open-Source Python Library for Materials Analysis. *Comput. Mater. Sci.* **2013**, *68*, 314–319. https://doi.org/10.1016/j.commatsci.2012.10.028.

(4) Zhao, Y.; Truhlar, D. G. The M06 Suite of Density Functionals for Main Group Thermochemistry, Thermochemical Kinetics, Noncovalent Interactions, Excited States, and Transition Elements: Two New Functionals and Systematic Testing of Four M06-Class Functionals and 12 Other Function. *Theor. Chem. Acc.* **2008**, *120* (1–3), 215–241. https://doi.org/10.1007/s00214-007-0310-x.

(5) Marenich, A. V.; Cramer, C. J.; Truhlar, D. G. Universal Solvation Model Based on Solute Electron Density and on a Continuum Model of the Solvent Defined by the Bulk Dielectric Constant and Atomic Surface Tensions. *J. Phys. Chem. B* **2009**, *113* (18), 6378–6396. https://doi.org/10.1021/jp810292n.

(6) Grimme, S. Supramolecular Binding Thermodynamics by Dispersion-Corrected Density Functional Theory. *Chem. - A Eur. J.* **2012**, *18* (32), 9955–9964. https://doi.org/10.1002/chem.201200497.

(7) Grimme, S. Semiempirical GGA-Type Density Functional Constructed with a Long-Range Dispersion Correction. *J. Comput. Chem.* **2006**, *27* (15), 1787–1799. https://doi.org/10.1002/jcc.20495.

(8) Warlin, N.; Nilsson, E.; Guo, Z.; Mankar, S. V.; Valsange, N. G.; Rehnberg, N.; Lundmark, S.; Jannasch, P.; Zhang, B. Synthesis and Melt-Spinning of Partly Bio-Based Thermoplastic Poly(Cycloacetal-Urethane)s toward Sustainable Textiles. *Polym. Chem.* **2021**, *12* (34), 4942–4953. https://doi.org/10.1039/D1PY00450F.

(9) Kultys, A.; Rogulska, M.; Głuchowska, H. The Effect of Soft-Segment Structure on the Properties of Novel Thermoplastic Polyurethane Elastomers Based on an Unconventional Chain Extender. *Polym. Int.* **2011**, *60* (4), 652–659. https://doi.org/10.1002/pi.2998.

(10) Govorčin Bajsić, E.; Rek, V. Thermal Stability of Polyurethane Elastomers before and after UV Irradiation. *J. Appl. Polym. Sci.* **2001**, *79* (5), 864–873. https://doi.org/10.1002/1097-4628(20010131)79:5<864::AID-APP110>3.0.CO;2-D.

(11) Du, Y.; Zhang, J.; Zhou, C. Synthesis and Properties of Waterborne Polyurethane-Based PTMG and PDMS as Soft Segment. *Polym. Bull.* **2016**, *73* (1), 293–308. https://doi.org/10.1007/s00289-015-1487-0.
