# Supplementary material for: Circular Polydiketoenamine Elastomers with Exceptional Creep Resistance via Multivalent Cross-Linker Design
Source: ACS Cent Sci. 2023 Nov 17;10(1):54–64. doi: 10.1021/acscentsci.3c01096 (PMC10823519; doi:10.1021/acscentsci.3c01096)
Supplement: Supplementary file 2 — oc3c01096_si_002.pdf [file oc3c01096_si_002.pdf]

oc-2023-01096d.R1

Name: Peer Review Information for "Circular Polydiketoenamine Elastomers with Exceptional Creep Resistance via Multivalent Crosslinker Design"

First Round of Reviewer Comments

Reviewer: 1

Comments to the Author

This manuscript reports the synthesis, characterization and recycling of two chemically similar but topologically dissimilar polydiketoenamine (PDK) elastomers. Through the stress relaxation and the creep experiments, the authors demonstrate excellent creep resistance for both the filled and unfilled PDK-multivalent elastomers, in contrast to their monovalent counterparts. The authors correlate such difference to the better performance of 4-arm crosslinkers versus 3-arm ones in suppressing the long-ranged terminal flow. The authors also report a significant difference in depolymerization properties of these two elastomers, which the authors attribute to the difference in the transition state free energy. Form a practical point of view, these findings are interesting and useful; however, there is an overall lack of firm evidence that the topological difference in the crosslinker structures independently leads to the observed rheological difference. Based on the synthetic approach (page 10 of the SI) provided by the authors, the reviewer did some brief calculations and found that the number density of the crosslinking points (Y-junctions in both cases) in the PDK-monovalent is only ~ 1/3 of the PDK-multivalent. Therefore, the reviewer suspects that the crosslinking density is the main factor unaccounted for in the current manuscript, and its effect on the network elasticity of the materials is non-trivial.

In order to justify the direct comparison between PDK-multivalent and PDK-monovalent elastomers, the authors have to at least do the followings:

(1) Prove that the number densities of the crosslinks are equal or similar in the two cases.

(2) Measure the gel and sol fractions of the two materials (unfilled).

As one can clearly see from the frequency sweep (Figure 2a and 2d) of the two unfilled elastomers, the PDK-multivalent has a gel-like behavior (G’>>G’’ irrespective of frequency), while the PDK-monovalent has a typical liquid-like behavior. This single observation puts a big question mark on the comparison between the two materials, and can easily nullify the conclusions derived therein.

Although the manuscript presented certain novelty arising from the synthesis and the unexpected difference in the recyclability of the two elastomers, it apparently does not fit the standard of ACS Central Science in current status. Thus, the article is better suited to more specific journals, such as Macromolecules or ACS Sustainable Chemistry and Engineering.

Reviewer: 2

Comments to the Author

This is an excellent comprehensive article from Helms and co-workers that details the discovery of creep resistant PDK elastomers that are also chemically recyclable. The combination of mechanistic studies alongside mechanical testing data provides for a great explanation of how short range vs long range chain mobility is drastically impacted at the molecular level. I recommend publication in ACS CS after minor changes noted below:

1. Figure text size could be larger to increase readability

2. Thermal stability (p 13 – 14): isothermal data at 150C is given for multivalent formulations but no monovalent formulations. How does monovalent compare?

3. Figure 6/S11: I recommend using chemical structures (S11) on the potential energy curve in the main text (Fig 6). It is difficult to follow the structures as currently depicted in Fig 6.

Reviewer: 3

Comments to the Author

This manuscript describes the synthesis and characterization of two distinct variants of polydiketoenamine (PDK) elastomers which function as covalent adaptable networks enabled by thermally activated dynamic bond exchange at crosslinking sites. The main difference between the two variants is the nature of the crosslinker: the “multivalent” elastomer possesses ditopic crosslink functionality at the chain ends of a flexible pTHF chain, while the “monovalent” elastomer features monotopic linkages at the end of a flexible pTHF chain and is crosslinked by a tri-topic small molecule (TREN). Through systematic thermal characterization and mechanical testing (rheology), the authors demonstrate that the “multivalent” elastomers possess higher strength and lower creep than the “monovalent” elastomers, in unfilled samples and in carbon-filled composites. They also show that the multivalent elastomers undergo faster hydrolytic degradation than the monovalent elastomers, despite the monovalent elastomers possessing a lower number of covalent crosslinks. The authors’ interpretation of the rheological data distinguishes the relative contributions of physical and covalent crosslinking and how these contributions evolve during stress relaxation in the two variants. In achieving

a highly degradable, thermally processible elastomeric material that exhibits low creep, this work addresses a clear need for sustainable polymeric materials (especially elastomers) that possess good mechanical properties. The fundamental insights from this work regarding the design of polymer architectures that give rise to these properties also stands to be broadly applicable in other systems.

This work is well written and I believe the conclusions are well-supported by data (for the most part). I am in favor of publishing this manuscript, pending clarification on several important experimental details. The main items currently lacking that I feel are important to the central conclusions of the paper are: a) The molecular weight of the pTHF-diamine used in the monovalent elastomers, and b) A clear description of how the molar ratio of amines to triketones was determined for both variants.

Regarding point a): my understanding of the authors' interpretation of the rheological data is that it relies on the pTHF-diamine and the pTHF-TREN having very similar molecular weight characteristics (Mn and dispersity). If the pTHF-diamine has higher MW or dispersity than the pTHF-TREN, I believe a more cautious interpretation of the physical vs. covalent cross-links distinction is warranted.

Regarding point b): the description in the main text referring to the construction of the monovalent elastomers, "we matched the pTHF weight fraction and total excess amine content to those featured in the PDK-multivalent elastomer networks." (pg. 5) is unclear, specifically what is meant by "matched the pTHF weight fraction," as well as how the amine content of the pTHF-diamine was determined. The supporting information does not provide the molecular weight of the pTHF-diamine (purchased from the manufacturer). The synthetic procedure indicates "pTHF-diamine (4.0 g, 2.35 mmol) and TK-10 (1.25 g, 2.80 mmol) were combined in a glass vial and heated to 110 ºC with stirring for 30 min until the mixture became homogeneous and evolution of bubbles ceased. The melt was cooled to 60 ºC, and TREN (0.1 g, 0.68 mmol) was added rapidly…" For the pTHF-diamine, it is important to know what "2.35 mmol" refers to (i.e., does this refer to mol pTHF-diamine, or does it refer to mol amines in pTHF-diamine, which is twice the former), and how it was determined. If the MW is unknown, I recommend MALDI and NMR analysis. The same goes for the synthetic procedures for the multivalent elastomer – what do the molar values refer to, and if they refer to the mol primary amines, was that calculated from proton NMR or otherwise? These details are key to verifying that the molar ratio of primary amines to triketones is 1.3:1 across all samples. When I attempted to calculate these ratios, assuming the molar values refer to mol polymer, I obtained a ratio of 1.45:1 for the multivalent and 1.20:1 for the monovalent, rather than 1.3.

One other experimental detail that detracts slightly from the ability to make a clean comparison between these systems is the fact that the multivalent elastomers were crosslinked in a THF solution, but the monovalent elastomers were crosslinked in a solvent-free melt. I am not sure how this might impact the morphology of the network, but it seems like it could if there is some phase separation in the melt when the crosslinker (TREN) is introduced. It would be beneficial for the authors to provide discussion or relevant evidence that the properties of these types of elastomers do not greatly depend on melt vs. solvent-based crosslinking.

Author's Response to Peer Review Comments:

Editor: Please include the email address of the corresponding author on the first page of the manuscript, and the Supporting Information if submitted, with an asterisk next to their name in the author list. Please be sure to label "email."

Author Response: Corresponding author email has been added to page 2 of the manuscript and page S2 of the SI. Corresponding author has been denoted with asterisk on page 1 of the manuscript and page S1 of the SI.

Editor: TOC/SYNOPSIS: Please move to the last page of the manuscript, beneath the References.

Author Response: TOC and synopsis have been moved as requested and appears on page 27 beneath the references.

Editor: SI PG#S: The supporting information pages must be numbered consecutively, starting with page S1.

Author Response: SI page numbers have been updated to be consecutive starting with S1.

Reviewer 1: This manuscript reports the synthesis, characterization and recycling of two chemically similar but topologically dissimilar polydiketoenamine (PDK) elastomers. Through the stress relaxation and the creep experiments, the authors demonstrate excellent creep resistance for both the filled and unfilled PDK-multivalent elastomers, in contrast to their monovalent counterparts. The authors correlate such difference to the better performance of 4-arm crosslinkers versus 3-arm ones in suppressing the longranged terminal flow. The authors also report a significant difference in depolymerization properties of these two elastomers, which the authors attribute to the difference in the transition state free energy. Form a practical point of view, these findings are interesting and useful; however, there is an overall lack of firm evidence that the topological difference in the crosslinker structures independently leads to the observed rheological difference. Based on the synthetic approach (page 10 of the SI) provided by the authors, the reviewer did some brief calculations and found that the number density of the crosslinking points (Y-junctions in both cases) in the PDK-monovalent is only ~ 1/3 of the PDK-multivalent. Therefore, the reviewer suspects that the crosslinking density is the main factor unaccounted for in the current manuscript, and its effect on the network elasticity of the materials is non-trivial.

In order to justify the direct comparison between PDK-multivalent and PDK-monovalent elastomers, the authors have to at least do the followings:

(1) Prove that the number densities of the crosslinks are equal or similar in the two cases.

Author Response: We appreciate the careful examination of the expect crosslinking structure and agree that the effect on elasticity is critical to our conclusions. To this end, we calculated the crosslink density for both formulations, n , as $\nu =$ "___#! ! where $M_\$ =$ ___%&'(" .

We measured the bulk density of both formulations to obtain r = 1.15 g mL^{-1} for PDKmonovalent and r = 1.11 g mL^{-1} for PDK-multivalent. The values for T and G' are taken from the amplitude sweep at 30 ºC, and R is the gas constant. From these calculations we obtained:

n = 3.32×10^{-5} mol g^{-1} for PDK-monovalent

n = 3.59×10^{-5} mol g^{-1} for PDK-multivalent

PDK-multivalent has a slightly higher molar density of crosslinks as expected from the shear modulus: ≈7% greater than PDK-monovalent. However, given the higher expected density of covalent crosslinks compared to PDK-monovalent, this data supports our conclusion that PDK-monovalent must contain significant non-covalent entanglements to produce the observed shear modulus and crosslinking density.

We have updated the manuscript with this data to further support our discussion.

We included the following passage on page 8:

We calculated the crosslinking density for both formulations from the equation:

$\nu =$ ___! (3)

"#! where

$M_\$ =$ %&'___((4)

Here, r is bulk density, *R* is the gas constant, *T* is absolute temperature, and *G* is the shear storage modulus at 30 ºC. We obtain n = 3.32×10^{-5} mol g^{-1} for PDK-monovalent and n = 3.59×10^{-5} mol g^{-1} for PDK-multivalent, confirming comparable crosslinking density for both formulations.

We included the following passage on page S11 of the SI:

Bulk density measurements.
The bulk density of the crosslinked elastomers was measured using the density bottle method. The empty mass of an oven-dried density bottle with capillary stopper was recorded (m_1) and loaded with a

pressed elastomer sample that was cut into a 8 mm x 1 mm disc. Sample mass was recorded (m_2). The bottle was then filled with DI water that was equilibrated at 20 ºC and the total mass was recorded (m_3). Finally, the sample was removed and the bottle was refilled with DI water and the mass was recorded (m_4). The specific gravity of each sample was determined from the equation $g = \frac{)\#*)\$}{()\#*)\$)*()\%*)\&)}$ and the result was converted to density using the measured density of DI water at 20 ºC (0.998 g mL^{-1}). The calculated density for PDK-monovalent was 1.15 g mL^{-1} and the density for PDK-multivalent was 1.11 g mL^{-1}.

Reviewer 1: (2) Measure the gel and sol fractions of the two materials (unfilled).

Author Response: We measured the gel fraction of both unfilled materials and obtained:

PDK-multivalent = 96.3% and PDK-monovalent = 94.1%.

We expect that this difference does not have a significant effect on our rheology results.

We have updated the manuscript on page 6 with the sentence:

“This method produced a high gel fraction for the monovalent and multivalent formulations (94.1% and 96.3% respectively).”

We added this section to the SI on page S11:

Gel fraction measurements.

Elastomer samples were pressed as described above, cut into 8 mm x 1 mm discs with a biopsy punch, and weighed. Samples were incubated in 4 mL chloroform at room temperature for 48 h. Chloroform was exchanged 8 times over this period. Samples were then dried to constant mass under vacuum and the final mass was recorded. The gel fraction for PDK-monovalent was 94.1% and the gel fraction of PDK-multivalent was 96.3%.

Reviewer 1: As one can clearly see from the frequency sweep (Figure 2a and 2d) of the two unfilled elastomers, the PDK-multivalent has a gel-like behavior (G’>>G’’ irrespective of frequency), while the PDK-monovalent has a typical liquid-like behavior. This single observation puts a big question mark on the comparison between the two materials, and can easily nullify the conclusions derived therein.

Author Response: We agree that these formulations must be compared under appropriate rheological conditions. We indeed observe $G^{"}>G^{'}$ in PDK-monovalent, but only one instance at low frequency (≈ 0.3-0.4 rad s^{-1}) and 150 ºC. This observation is consistent with our discussion of how mono- vs. multivalent chain ends affects chain mobility at elevated temperature. We selected 10 rad s^{-1} for our amplitude sweep experiments to maintain $G^{'}>>G^{"}$ for all temperatures, allowing us to compare both formulations under conditions where they behave as viscoelastic solids.

Reviewer 2: This is an excellent comprehensive article from Helms and co-workers that details the discovery of creep resistant PDK elastomers that are also chemically recyclable. The combination of mechanistic studies alongside mechanical testing data provides for a great explanation of how short range vs long range chain mobility is drastically impacted at the molecular level. I recommend publication in ACS CS after minor changes noted below:

Author Response: We appreciate the enthusiastic review and have made changes as described under each point below.

Reviewer 2: Figure text size could be larger to increase readability

Author Response: We have increased legend and axis text size in figures 2 (page 7), 3 (page 12), and 4 (page 13).

Reviewer 2: Thermal stability (p 13 – 14): isothermal data at 150C is given for multivalent formulations but no monovalent formulations. How does monovalent compare?

Author Response: We performed the isothermal TGA measurement for PDKmonovalent and found both monovalent and multivalent formulations have <1% mass loss at 10,000 seconds.

We updated the following sentence in the manuscript on page 14:

We measured <1% mass loss in both PDK-multivalent (Figure S6) and PDK-monovalent (Figure S7) with or without carbon black after 10,000 s at 150 ºC by TGA, verifying excellent thermal stability at the highest temperature in our rheological experiments.

We included the TGA data as Figure S7 on page S20 of the SI.

Reviewer 2: Figure 6/S11: I recommend using chemical structures (S11) on the potential energy curve in the main text (Fig 6). It is difficult to follow the structures as currently depicted in Fig 6.

Author Response: We have moved the chemical structures in Figure S11 to the main text as Figures 6A and 6B on page 6 to clarify the small molecule configurations along the reaction coordinate.

Reviewer 3: This manuscript describes the synthesis and characterization of two distinct variants of polydiketoenamine (PDK) elastomers which function as covalent adaptable networks enabled by thermally activated dynamic bond exchange at crosslinking sites. The main difference between the two variants is the nature of the crosslinker: the "multivalent" elastomer possesses ditopic crosslink functionality at the chain ends of a flexible pTHF chain, while the "monovalent" elastomer features monotopic linkages at the end of a flexible pTHF chain and is crosslinked by a tri-topic small molecule (TREN). Through systematic thermal characterization and mechanical testing (rheology), the authors demonstrate that the "multivalent" elastomers possess higher strength and lower creep than the "monovalent" elastomers, in unfilled samples and in carbon-filled composites. They also show that the multivalent elastomers undergo faster hydrolytic degradation than the monovalent elastomers, despite the monovalent elastomers possessing a lower number of covalent crosslinks. The authors' interpretation of the rheological data distinguishes the relative contributions of physical and covalent crosslinking and how these contributions evolve during stress relaxation in the two variants. In achieving a highly degradable, thermally processible elastomeric material that exhibits low creep, this work addresses a clear need for sustainable polymeric materials (especially elastomers) that possess good mechanical properties. The fundamental insights from this work regarding the design of polymer architectures that give rise to these properties also stands to be broadly applicable in other systems.

This work is well written and I believe the conclusions are well-supported by data (for the most part). I am in favor of publishing this manuscript, pending clarification on several important experimental details. The main items currently lacking that I feel are important to the central conclusions of the paper are: a) The molecular weight of the pTHF-diamine used in the monovalent elastomers, and b) A clear description of how the molar ratio of amines to triketones was determined for both variants.

Author Response: We appreciate the detailed and thoughtful review and careful inspection of our manuscript. A discussion of both points is provided below.

Reviewer 3: Regarding point a): my understanding of the authors' interpretation of the rheological data is that it relies on the pTHF-diamine and the pTHF-TREN having very similar molecular weight characteristics (Mn and dispersity). If the pTHF-diamine has higher MW or dispersity than the pTHF-TREN, I believe a more cautious interpretation of the physical vs. covalent cross-links distinction is warranted.

Author Response: This is an important aspect of these formulations. We measured M_n and M_w via MALDI and obtained M_n = 1,535 g mol^{-1} and PDI = 1.21 for pTHF-diamine, and M_n = 2,203 g mol^{-1} and Đ = 1.07 for pTHF-TREN, confirming that pTHF-diamine is lower M_n than pTHF-bis-TREN.

We believe the more critical factor here arises from the lower covalent crosslinking density for the monovalent formulation. We pre-react pTHF-diamine with TK-10 before adding TREN to crosslink, which we predicted would also generate linear segments via addition polymerization between pTHF-diamine and TK-10. Since we unexpectedly observed a similar crosslinking density between the formulations at 30 ºC, we concluded that the noncovalent entanglements in the pTHF-monovalent linear segments contributed significantly to the modulus. The presence of the non-covalent entanglements also contributes to the divergent trends in modulus vs. temperature between the monovalent and multivalent formulations.

We added MALDI measurements for pTHF-diamine M_n and Đ to page S3 of the SI as follows:

Poly(tetrahydrofuran) diamine (pTHF-diamine) was received from Huntsman Chemical, and we calculated Mn = 1,535 g mol^{-1} and Đ = 1.21 via MALDI.

We added MALDI measurements for pTHF-bis-TREN M_n and Đ to page S8 of the SI as follows:

M_n = 2203 g mol^{-1}, Đ = 1.07 (MALDI).

Reviewer 3: Regarding point b): the description in the main text referring to the construction of the monovalent elastomers, “we matched the pTHF weight fraction and total excess amine content to those featured in the PDK-multivalent elastomer networks.” (pg. 5) is unclear, specifically what is meant by “matched the pTHF weight fraction,” as well as how the amine content of the pTHF-diamine was determined. The supporting information does not provide the molecular weight of the pTHF-diamine (purchased from the manufacturer). The synthetic procedure indicates “pTHF-diamine (4.0 g, 2.35 mmol) and TK-10 (1.25 g, 2.80 mmol) were combined in a glass vial and heated to 110 ºC with stirring for 30 min until the mixture became homogeneous and evolution of bubbles ceased. The melt was cooled to 60 ºC, and TREN (0.1 g, 0.68 mmol) was added rapidly…” For the pTHF-diamine, it is important to know what “2.35 mmol” refers to (i.e., does this refer to mol pTHF-diamine, or does it refer to mol amines in pTHF-diamine, which is twice the former), and how it was determined. If the MW is unknown, I recommend MALDI and NMR analysis. The same goes for the synthetic procedures for the multivalent elastomer – what do the molar values refer to, and if they refer to the mol primary amines, was that calculated from proton NMR or otherwise? These details are key to verifying that the molar ratio of primary amines to triketones is 1.3:1 across all samples. When I attempted to calculate these ratios, assuming the molar values refer to mol polymer, I obtained a ratio of 1.45:1 for the multivalent and 1.20:1 for the monovalent, rather than 1.3.

Author Response: We appreciate the reviewer's attention to detail and we are happy to clarify and elaborate on these points. We match the mass fraction of the pTHF component for the monovalent and multivalent formulations. We report mol of pTHF-diamine or pTHF-TREN based on the average molecular weight of the pTHF macromer, rather than mol of amine. This does require clarification on one point: since we need to add a small amount of TREN as a crosslinker in the monovalent formulation, the pTHF mass fraction cannot be exactly matched and is slightly lower relative to the multivalent formulation. The full calculation is detailed below.

Using M_n = 2,203 g mol^{-1} for pTHF-bis-TREN and 4 primary amines per macromer, we calculate 1 g pTHF-bis-TREN has 1.82 mmol amines (1/2203 * 4). An amine:triketone ratio of 1.3:1 thus requires 1.4 mmol triketones. TK-10 has molar mass 446.27 g mol^{-1} and 2 triketones per molecule. Thus,

$1.4 * 10^{*-} mol \quad triketone *$ ______________ ../.'$ 27 g mol

"

$mol\ triketone\ per\ mol\ TK-10$

yields 0.312 g TK-10. This matches our methods in the SI where we report 1 g pTHF-bisTREN and 0.31 g TK-10. This also gives us a pTHF weight fraction of 0.76 (1/1.31).

Similarly, we use M_n = 1,535 g mol^{-1} for pTHF-diamine and 2 primary amines per macromer. Using 4 g pTHF-diamine and 1.25 g TK-10 we again get a pTHF mass fraction of 0.76. However, it is necessary to add TREN as a crosslinker. 1.25 g TK-10 contributes

5.6 mmol triketones. 4 g pTHF-diamine contributes 5.2 mm amines (this corresponds to 2.6 mmol pTHF-diamine, not 2.35 as we reported originally; the SI has been corrected). To get a 1.3:1 ratio of amine:triketone we need 7.3 mmol amines total, so we add 2.1 mmol primary amines via TREN. This requires 0.1 g TREN (0.69 mmol, which contains 2.1 mmol primary amines). This gives us a total pTHF weight fraction of 0.75, which is ≈1.3% lower than the multivalent formulation.

We acknowledge that these calculations are based on number-average molecular weights that have some dispersity, as well as an assumption of 100% chain-end functionalization. However, we maintain that our reported calculations are consistent throughout for a 1.3:1 amine:triketone ratio, and that we have matched the pTHF weight fractions as closely as possible given the formulation requirements.

We updated the manuscript with the following sentence on page 5:

We matched the pTHF weight fraction between the two formulations as closely as possible (75% w/w for PDK-monovalent vs. 76% w/w for PDK-multivalent), and again set the total amine-to-triketone molar ratio to 1.3:1 for PDK-monovalent.

We updated the SI on page S11 in the sections "Synthesis of PDK-multivalent elastomers" and "Synthesis of PDK-monovalent elastomers" to specify the moles of functional groups for pTHF-bis-TREN, pTHF-diamine, TK-10, and TREN. We also corrected the quantity of pTHF-diamine in mmol.

Reviewer 3: One other experimental detail that detracts slightly from the ability to make a clean comparison between these systems is the fact that the multivalent elastomers were crosslinked in a THF solution, but the monovalent elastomers were crosslinked in a solvent-free melt. I am not sure how this might impact the morphology of the network, but it seems like it could if there is some phase separation in the melt when the crosslinker (TREN) is introduced. It would be beneficial for the authors to provide discussion or relevant evidence that the properties of these types of elastomers do not greatly depend on melt vs. solvent-based crosslinking.

Author Response: This is an important point. An in-depth investigation into the evolution of phase structure during polymerization is likely beyond the scope of this article. However, both triketone and pTHF components can form crystalline domains, and our DSC data shows no thermal transitions that could correspond to crystallinity in a phasesegregated structure. We only observe microcrystalline structure in PDK-monovalent below 20 ºC (Figure S3), which is below the temperature range of our rheological experiments. It's also possible that the compression molding produces a single-phase material, e.g., if any separation did occur during the initial polymerization. We expect phase structure to become critical in reinforced rubbers, particularly those with high weight or volume fraction of filler, and anticipate studying this important phenomenon in more detail in future work.
